# Baricitinib Augments Lonafarnib Therapy to Preserve Colonic Homeostasis and Microbial Balance in a Mouse Model of Progeria

**DOI:** 10.1111/acel.70273

**Published:** 2025-10-20

**Authors:** Moritz Schroll, Yacine Amar, Peter Krüger, Klaus Neuhaus, Karima Djabali

**Affiliations:** ^1^ Epigenetics of Aging, Department of Dermatology and Allergy, TUM School of Medicine and Health, Munich Institute of Biomedical Engineering Technical University of Munich Garching Germany; ^2^ Department of Dermatology and Allergy, School of Medicine Technical University of Munich Munich Germany; ^3^ Core Facility Microbiome, ZIEL‐Institute for Food & Health Technical University of Munich Freising Germany

**Keywords:** Baricitinib, Hutchinson‐Gilford progeria syndrome; JAK–STAT, lonafarnib, microbiome

## Abstract

Hutchinson‐Gilford Progeria Syndrome (HGPS) is a fatal genetic disorder caused by progerin, a mutant lamin A variant that disrupts nuclear architecture and drives systemic cellular dysfunction. Gastrointestinal (GI) involvement in HGPS remains poorly understood, despite growing evidence of gut abnormalities and microbial dysbiosis in progeroid mouse models. Here, we provide the first comprehensive characterization of colonic pathology in *Lmna* ^*G609G/G609G*^ mice and assess the therapeutic impact of baricitinib (Bar), a JAK–STAT inhibitor, lonafarnib (FTI), the only FDA‐approved therapy, and their combination on colonic health. Bar + FTI combination therapy most effectively lowered progerin levels, preserved colonic architecture and epithelial regeneration markers, while also reducing inflammation, cellular senescence, and early fibrotic changes. Notably, FTI monotherapy aggravated inflammation via STAT1 activation, an effect reversed by Bar co‐administration. Bar also emerged as the primary driver in mitigating colonic tissue senescence, highlighting its role in supporting intestinal homeostasis. In addition, we observed marked microbial dysbiosis in HGPS mice, particularly in late‐stage disease. While both monotherapies induced distinct shifts in gut microbiota, combination therapy preserved a profile more closely resembling healthy controls. These findings expand the current understanding of GI involvement in HGPS and identify the colon as a site where JAK–STAT inhibition enhances the therapeutic profile of FTI.

## Graphical abstract

Combined baricitinib and lonafarnib treatment improved colonic pathology in *Lmna* ^*G609G/G609G*^ progeria mice by reducing progerin, maintaining epithelial regeneration, mitigating inflammation and senescence, and preserving microbiome composition.

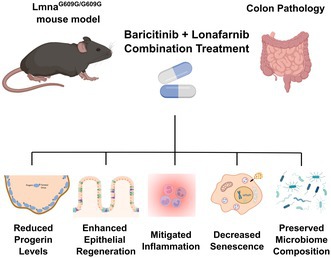

## 1 Introduction

Hutchinson‐Gilford progeria syndrome (HGPS; OMIM #176670) is a rare and fatal genetic disorder characterized by premature aging. As of 2024, 149 cases were reported globally, with an estimated prevalence of 1 in 20 million (Progeria Research Foundation 2024). HGPS is primarily caused by a de novo *LMNA* mutation (c.1824C>T; G608G), resulting in progerin, a permanently farnesylated lamin A variant (De Sandre‐Giovannoli et al. 2003; Eriksson et al. 2003; Goldman et al. 2004). Progerin accumulation disrupts the nuclear architecture and triggers multiple cellular dysfunctions, including DNA damage, altered gene expression, mitochondrial defects, and premature senescence (Cao et al. 2011; Goldman et al. 2004; Scaffidi and Misteli 2005; Shumaker et al. 2006). Clinically, HGPS manifests with symptoms such as alopecia, lipodystrophy, growth retardation, and severe atherosclerosis, leading to an average life expectancy of 14.6 years (Gordon et al. 2014; Merideth et al. 2008). Therapeutic options remain limited. Lonafarnib, a farnesyltransferase inhibitor (FTI), is currently the only FDA‐approved therapy (US Food and Drug Administration 2020). By blocking progerin farnesylation, FTI reduces aberrant accumulation of the toxic progerin, thereby slowing disease progression and extending mean survival by 1.6 years (Capell et al. 2005; Gordon et al. 2014). Recent studies have shown that targeting inflammatory pathways alleviates key cellular and tissue‐level abnormalities in HGPS, underscoring inflammation as a modifiable driver of disease progression (Liu et al. 2019; Muela‐Zarzuela et al. 2024; Osorio et al. 2012; Squarzoni et al. 2021). Baricitinib (Bar), a JAK–STAT inhibitor approved for several inflammatory conditions, restored cellular homeostasis and suppressed inflammatory signaling in HGPS fibroblasts (Arnold et al. 2021; European Medicines Agency 2023; Liu et al. 2019). In combination with FTI, Bar further improved healthspan and extended lifespan in an HGPS mouse model (Krüger et al. 2025).

While much of the research in HGPS has focused on cardiovascular defects, emerging evidence in progeroid mouse models suggests that HGPS is also associated with gastrointestinal (GI) dysfunction and gut microbiota imbalance (Barcena et al. 2018, 2019; Hou et al. 2023; Kreienkamp et al. 2019; Wang et al. 2024; Zhang, Hu, et al. 2023). Notably, fecal microbiota transplantation from healthy mice improved healthspan and survival in these models (Barcena et al. 2019). Structural abnormalities have been documented throughout the GI tract, including shortened colon length, fibrotic esophageal tissue and gastric mucosal atrophy (Barcena et al. 2018; Kreienkamp et al. 2019; Wang et al. 2024; Zhang, Hu, et al. 2023). Although these findings point to broader GI involvement, the colon remains largely unexamined in HGPS, despite being a key site of immune coordination, host–microbe interaction and bidirectional gut‐brain communication (Choi and Augenlicht 2024; Cryan et al. 2019; Powell et al. 2017). While FTI provides clinical benefits to HGPS patients, its impact on gut health and the microbiome remains unknown (Gordon et al. 2014; US Food and Drug Administration 2020). Importantly, GI symptoms are the most frequently reported side effects (Eiger BioPharmaceuticals Inc. 2020). In contrast, JAK–STAT inhibitors are well‐established treatments for inflammatory bowel diseases (IBDs), which are also characterized by inflammation and gut microbial dysbiosis (Herrera‐deGuise et al. 2023; Honap et al. 2024). Given these parallels, JAK–STAT inhibition may offer a targeted strategy for mitigating GI dysfunction in HGPS.

In this study, we present a comprehensive analysis of colon‐specific pathology in the Lmna^G609G/G609G^ progeroid mice and investigate how it is modulated by Bar, FTI, and their combination (Osorio et al. 2011). We show that combined Bar + FTI treatment alleviates key pathological features, including disruption of mucosal architecture and epithelial renewal, inflammation, and cellular senescence. Notably, Bar counteracted FTI‐induced intestinal inflammation and contributed to a reduction in senescent cell burden. Additionally, combination treatment preserved a more balanced microbial profile compared to either monotherapy. These findings reveal previously unrecognized colonic involvement in HGPS and support JAK–STAT inhibition as a strategy to enhance the therapeutic profile of FTI in the GI tract.

## 2 Results

### 2.1 Bar + FTI Combination Treatment Alleviates Progerin Accumulation, Improves Colon Morphology and Partially Preserves Epithelial Regeneration in HGPS

**FIGURE 1:**
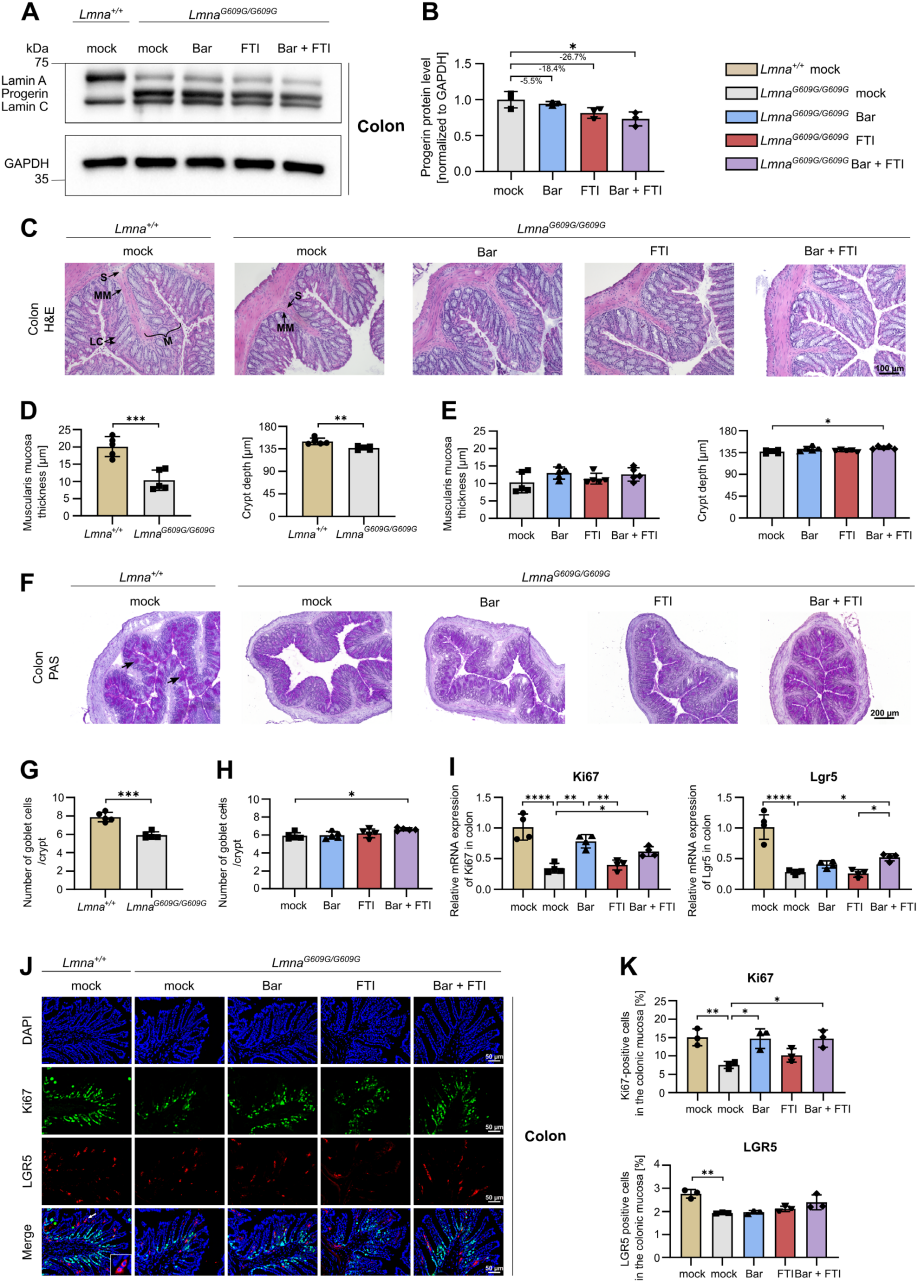
Combination treatment of Bar + FTI reduces progerin and partially preserves intestinal health in HGPS mice. (A) Western blot (WB) image of Lamin A, progerin and Lamin C protein expression in colonic tissue from *Lmna* ^*+/+*^ mock, *Lmna* ^*G609G/G609G*^ mock, Bar, FTI and Bar + FTI treated mice. GAPDH was used as a loading control. (B) Densitometric quantification of progerin protein levels in *Lmna* ^*G609G/G609G*^ mice based on WB data (*n* = 3). (C) Representative Hematoxylin and Eosin (H&E) stained images of colon tissue sections from *Lmna* ^*+/+*^ mock, *Lmna* ^*G609G/G609G*^ mock, Bar, FTI and Bar + FTI treated mice (mucosa (M), muscularis mucosa (MM), submucosa (S), intestinal crypts of Lieberkühn (LC)), (scale bar, 100 μm). (D) Muscularis mucosa thickness and Lieberkühn crypt depth in *Lmna* ^*+/+*^ mock and *Lmna* ^*G609G/G609G*^ mock mice (*n* = 5). (E) Muscularis mucosa thickness and Lieberkühn crypt depth in treated *Lmna* ^*G609G/G609G*^ mice (*n* = 5). (F) Representative periodic acid‐Schiff (PAS) stained images highlighting goblet cells in colonic tissue (magenta‐colored cells, black arrow) (scale bar, 200 μm). (G) Goblet cell count per crypt in *Lmna* ^*+/+*^ mock and *Lmna* ^*G609G/G609G*^ mock mice (*n* = 5). (H) Goblet cell count per crypt in treated Lmna^G609G/G609G^ mice (*n* = 5). (I) Analysis of mRNA levels of Ki67 and Lgr5 in colonic tissue across all groups (*n* = 4). (J) Immunofluorescence (IF) images of Ki67 (green) and LGR5 (red) expression in colon tissue of *Lmna* ^*+/+*^ mock, *Lmna* ^*G609G/G609G*^ mock, Bar, FTI and Bar + FTI treated mice. DAPI stain (blue) was used to counterstain nuclei (magnification 40; scale bar 50 μm). To show a detailed view of LGR5 expression, a zoom‐in box is provided for one representative group. The white arrow indicates the location of the zoomed region. (K) Percentage of Ki67‐positive and LGR5‐positive cells in the colonic mucosa based on IF analysis (*n* = 3). Data are expressed as the mean ± SD. *p < 0.05; **p < 0.01; ***p < 0.001 calculated using ordinary one‐way ANOVA followed by Tukey's Post Hoc test in *B*, *E*, *H*, *I*, and *K* and an unpaired *t*‐test in *D* and *G*.

To rule out differences in food intake as a confounder, we assessed the body weight across groups. No significant differences were observed among progeroid groups at Day 90, indicating comparable chow consumption (Figure S1). This is consistent with our previous findings, where body weight remained consistent across treatments throughout the lifespan (Krüger et al. 2025). The phenotype of HGPS is driven by the accumulation of progerin, whose expression varies across different tissues (Goldman et al. 2004; Kruger et al. 2024). To assess the effects of treatment on colonic progerin levels, we performed Western blot analysis. As expected, *Lmna* ^*+/+*^ mice (wildtype) lacked detectable progerin expression (Figure 1A). In *Lmna* ^*G609G/G609G*^ mice, FTI treatment led to a modest, non‐significant reduction in progerin levels (−18.4%, *p* = 0.103; Figure 1A,B). While Bar alone had a minimal effect (−5.5%), the combination treatment resulted in the most pronounced reduction (−26.7%, *p* = 0.019; Figure 1A,B). Within the colon, progerin was present across multiple compartments but was most abundant in the lamina propria of the mucosa and in the submucosa, with additional strong signal in the muscularis mucosa (Figure S2). To examine how progerin accumulation affects colonic morphology, we conducted histological analyses. Mock‐treated *Lmna* ^*G609G/G609G*^ mice exhibited a thinner muscularis mucosa and reduced crypt depth compared to *Lmna* ^*+/+*^ controls at 90 days of age, indicating compromised tissue structure and impaired regeneration (Figure 1C,D). While none of the treatments significantly influenced muscularis mucosa thickness, combination therapy substantially increased crypt depth (Figure 1C,E). PAS staining further demonstrated a marked decrease in goblet cell number in mock *Lmna* ^*G609G/G609G*^ mice compared to wildtype controls (Figure 1F,G). Goblet cells produce mucus that maintains epithelial integrity and barrier function, and their loss may weaken the protective mucus layer, increasing epithelial exposure to the gut microbiota and promoting mucosal inflammation (Gustafsson and Johansson 2022). Importantly, only the combination of Bar and FTI partially restored goblet cell numbers (Figure 1F,H). To investigate how these structural changes relate to intestinal function, we assessed markers of barrier integrity and epithelial regeneration. mRNA levels of tight junction genes were modestly downregulated in *Lmna* ^*G609G/G609G*^ mice, with little effect from treatments (Figure S3A–D). In contrast, key epithelial regeneration markers were significantly downregulated in mock‐treated *Lmna* ^*G609G/G609G*^ mice, including Ki67, a marker of epithelial proliferation, and Lgr5, a stem cell marker essential for intestinal renewal (Figure 1I). Bar alone and in combination with FTI increased Ki67 mRNA expression, while combination treatment partially restored Lgr5 expression (Figure 1I). FTI alone had no measurable effect on either marker. Immunofluorescence analysis confirmed a marked reduction in Ki67^+^ cells in the colonic mucosa of mock‐treated progeroid mice, which was effectively reversed by Bar and Bar + FTI treatment (Figure 1J,K). LGR5^+^ cell numbers were also reduced in mock *Lmna* ^*G609G/G609G*^ mice (1.9%) relative to wildtype (2.8%) and showed a moderate increase with combination treatment (2.4%; *p* = 0.0585) (Figure 1J,K). Together, these findings suggest that progerin accumulation is associated with reduced epithelial renewal in the colon and that JAK–STAT inhibition, particularly in combination with FTI, partially preserves stem and proliferative cell markers.

### 2.2 Bar Mitigates Colonic Inflammation and Counteracts FTI‐Induced Inflammation in HGPS Mice

**FIGURE 2:**
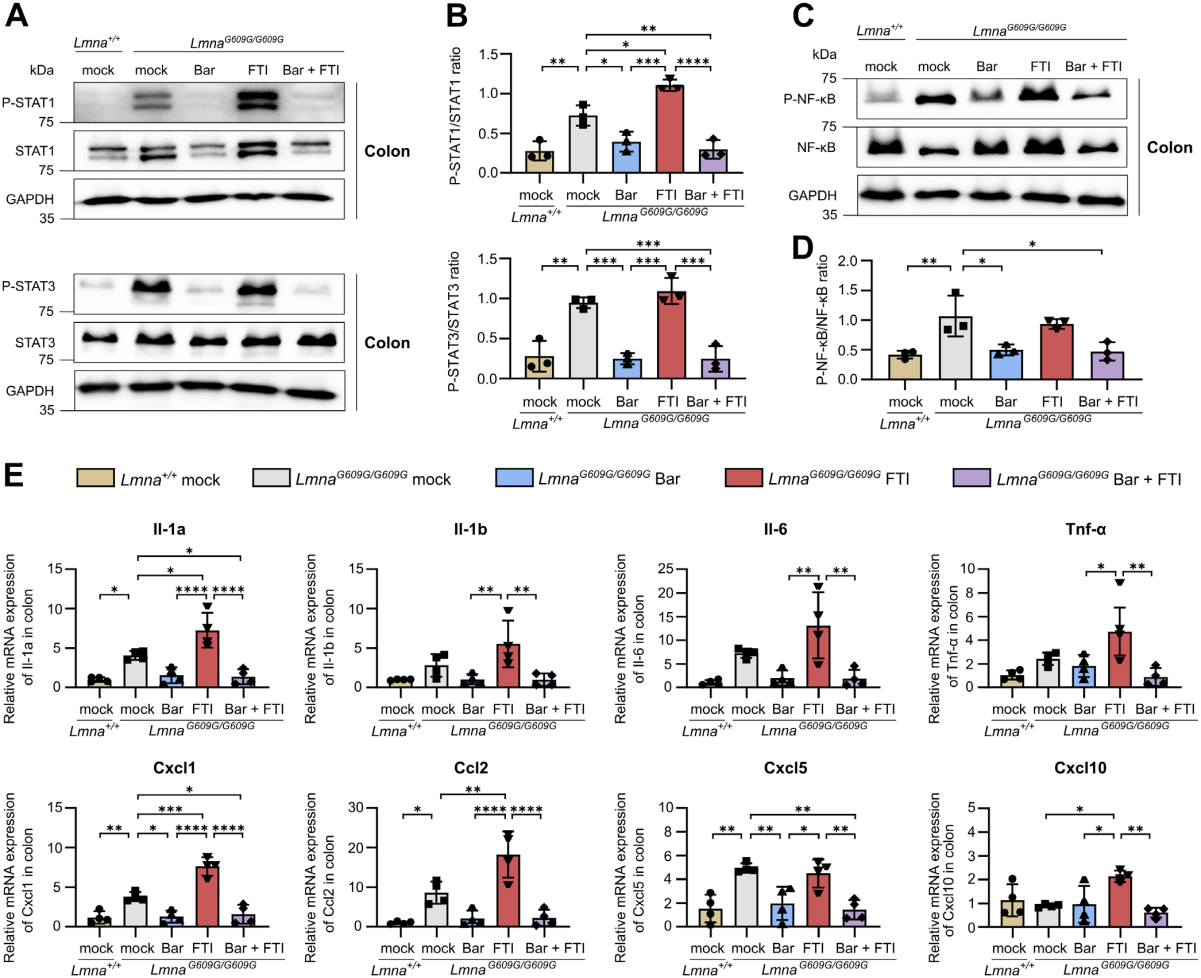
Bar treatment effectively suppresses colonic inflammation in HGPS mice, counteracting FTI. (A) WB images of P‐STAT1, STAT1, P‐STAT3, and STAT3 protein expression in colonic tissue from *Lmna* ^*+/+*^ mock, *Lmna* ^*G609G/G609G*^ mock, Bar, FTI, and Bar + FTI treated mice. GAPDH served as a loading control. (B) Densitometric quantification of P‐STAT1/STAT1 and P‐STAT3/STAT3 ratios based on WB data (*n* = 3). (C) WB images of P‐NF‐κB p65 and NF‐κB p65 protein expression in colonic tissue from all groups. GAPDH served as a loading control. (D) Densitometric quantification of P‐NF‐κB/NF‐κB ratios based on WB data (*n* = 3). (E) Analysis of mRNA levels of Il‐1a, Il‐1b, Il‐6, Tnf‐α, Cxcl1, Ccl2, Cxcl5, and Cxcl10 in total colon extracts of *Lmna* ^*+/+*^ mock, *Lmna* ^*G609G/G609G*^ mock, Bar, FTI, and Bar + FTI treated mice (*n* = 4). Data are expressed as the mean ± SD. *p < 0.05; **p < 0.01; ***p < 0.001; ****p < 0.0001 calculated using ordinary one‐way ANOVA followed by Tukey's Post Hoc test.

The JAK–STAT and NF‐κB pathways are key regulators of intestinal immune responses and are frequently dysregulated in IBDs, contributing to chronic mucosal inflammation and tissue damage (Herrera‐deGuise et al. 2023; Schreiber et al. 1998). Similar overactivation patterns have been observed in HGPS, where both pathways are consistently overactivated in fibroblasts and progeroid mouse models, reflecting a state of chronic systemic inflammation (Arnold et al. 2021; Cancado de Faria et al. 2023; Griveau et al. 2020; Liu et al. 2019; Osorio et al. 2012). However, whether these inflammatory pathways are similarly dysregulated in the GI tract of progeroid mice has not been previously examined. To address this, we assessed JAK–STAT and NF‐κB signaling in colon tissue from *Lmna* ^*G609G/G609G*^ mice. Western blot analysis revealed increased activation of STAT1 and STAT3 in mock‐treated progeroid mice compared to wildtype controls (Figure 2A,B). Notably, FTI monotherapy further increased STAT1 activation beyond levels seen in untreated progeroid mice, exacerbating the underlying inflammatory response (Figure 2A,B). Combined treatment with Bar reversed FTI‐induced STAT1 activation and also suppressed STAT3 hyperactivation observed in HGPS colon tissue (Figure 2A,B). As previously reported in HGPS, NF‐κB signaling was elevated in untreated *Lmna* ^*G609G/G609G*^ mice (Figure 2C,D) (Osorio et al. 2012). While FTI did not affect NF‐κB activation, Bar treatment, either alone or in combination with FTI, significantly reduced NF‐κB activity (Figure 2C,D). To examine the spatial localization of STAT1 pathway activation, we stained colonic tissue from *Lmna* ^*+/+*^ and *Lmna* ^*G609G/G609G*^ mice for IRF1, a direct and inducible transcriptional target of STAT1 (Abou El Hassan et al. 2017; Sekrecka et al. 2023). IRF1^+^ nuclei were confined to the mucosa, most enriched in the surface epithelium and upper crypt epithelium facing the lumen, with fewer positive cells deeper in the crypts and in the lamina propria (Figure S4). To assess downstream effects, we examined inflammatory cytokine expression. Consistent with previous studies, mock‐treated *Lmna* ^*G609G/G609G*^ mice exhibited elevated Il‐1a, Il‐1b, Il‐6, and Tnf‐α levels, which were further increased by FTI (Figure 2E) (Krüger et al. 2025; Muela‐Zarzuela et al. 2024; Squarzoni et al. 2021). In contrast, Bar treatment, alone or combined with FTI, restored cytokine levels to those observed in *Lmna* ^*+/+*^ mice. Similarly, chemokines Cxcl1, Ccl2, and Cxcl5 were elevated in mock‐treated *Lmna* ^*G609G/G609G*^ mice (Figure 2E). FTI further increased Cxcl1 and Ccl2 and induced Cxcl10 expression. These changes were reversed by Bar, alone or in combination with FTI, restoring levels to those seen in *Lmna* ^*+/+*^ mice. Additionally, occasional immune cell infiltration was observed in the colonic mucosa of mock‐treated *Lmna* ^*G609G/G609G*^ mice (Figure S5). However, this was not consistently present at the 90‐day time point, suggesting a progressive inflammatory phenotype. Collectively, these findings demonstrate that Bar mitigates colonic inflammation in HGPS by suppressing aberrant JAK–STAT and NF‐κB signaling and effectively counteracts the pro‐inflammatory activation induced by FTI.

### 2.3 Combination Treatment With Bar + FTI Attenuates Early Signs of Colon Fibrosis in HGPS Mice

**FIGURE 3:**
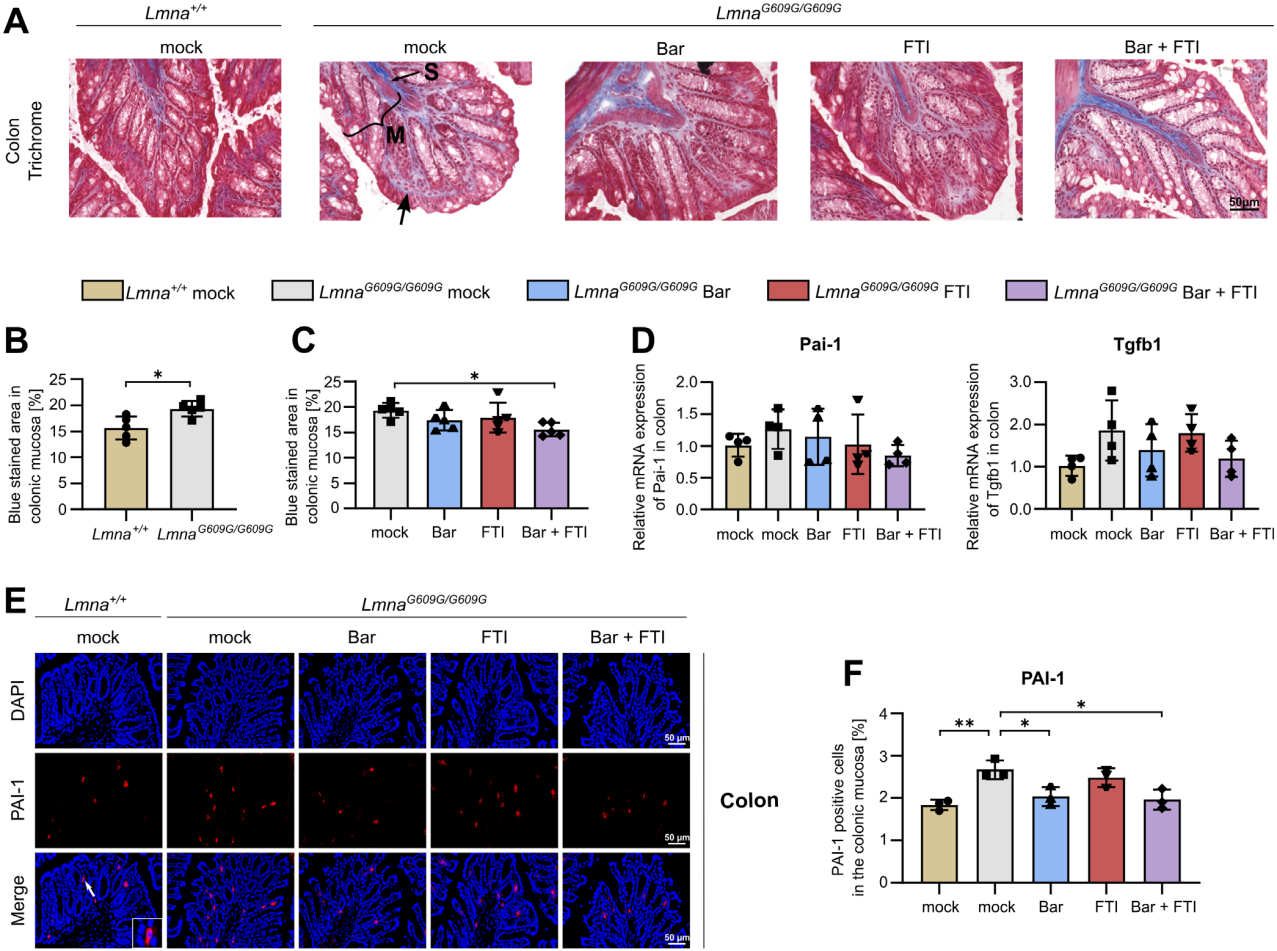
Combination treatment with Bar + FTI ameliorates early fibrotic features in the colon of HGPS mice. (A) Representative Masson's trichrome stained images of colon tissue sections from *Lmna* ^*+/+*^ mock, *Lmna* ^*G609G/G609G*^ mock, Bar, FTI or Bar + FTI treated mice. Black arrows show blue stained area (mucosa (M); submucosa (S); scale bar, 100 μm). (B) Blue stained area in the colonic mucosa in *Lmna* ^*+/+*^ mock and *Lmna* ^*G609G/G609G*^ mock mice (*n* = 5). (C) Blue‐stained area in the colonic mucosa in treated *Lmna* ^*G609G/G609G*^ mice (*n* = 5). (D) Analysis of mRNA levels of Pai‐1 and Tgfb1 in the colon of *Lmna* ^*+/+*^ mock, *Lmna* ^*G609G/G609G*^ mock, Bar, FTI or Bar + FTI treated mice (*n* = 4). (E) IF images of PAI‐1 expression (red) in colon tissue of *Lmna* ^*+/+*^ mock, *Lmna* ^*G609G/G609G*^ mock, Bar, FTI, or Bar + FTI treated mice. DAPI stain (blue) was used to counterstain nuclei (magnification 40; scale bar 50 μm). To show a detailed view of PAI‐1 expression, a zoom‐in box is provided for one representative group. The white arrow indicates the location of the zoomed region. (F) Percentage of PAI‐1 positive cells in the colonic mucosa based on IF analysis (*n* = 3). Data are expressed as the mean ± SD. *p < 0.05; **p < 0.01 calculated using ordinary one‐way ANOVA followed by Tukey's Post Hoc test in *C*, *D*, and *F* and an unpaired *t‐*test in *B*.

Given the established presence of inflammation in the colon, we next investigated whether progeroid mice exhibit signs of fibrotic remodeling, a common consequence of chronic GI inflammation (Fiocchi and Lund 2011). Notably, fibrosis in progeroid mice has been reported in multiple organs, suggesting systemic involvement (Hong et al. 2024; Kruger et al. 2024). In mock‐treated *Lmna* ^*G609G/G609G*^ mice, collagen accumulation was increased within the mucosal layer, with the most pronounced deposition observed above the crypts, near the luminal surface (Figure 3A,B). This is consistent with the previously observed reduction in crypt depth. In contrast, the submucosa and muscularis layers appeared unaffected, suggesting that fibrotic remodeling at this stage is restricted to the mucosa. Treatment with Bar or FTI alone did not significantly reduce collagen accumulation, indicating that single‐agent therapy may be insufficient to halt fibrotic progression (Figure 3A,C). In contrast, combination therapy with Bar + FTI markedly reduced collagen deposition, supporting its potential to delay fibrotic remodeling (Figure 3A,C). To further characterize the fibrotic response, we assessed fibrosis‐associated markers using immunofluorescence and qPCR. Vimentin expression showed a trend toward increased levels in the mucosa of mock‐treated progeroid mice, though this did not reach statistical significance (Figure S6A,B). α‐SMA staining revealed no evidence of myofibroblast activation, indicating an early fibrotic stage (Figure S6A). qPCR analysis of plasminogen activator inhibitor‐1 (Pai‐1) and Tgfb1 in total colonic extracts indicated a trend toward increased expression in mock *Lmna* ^*G609G/G609G*^ mice, though these differences did not reach statistical significance (Figure 3D). Given the established role of PAI‐1 in fibrosis and its emerging importance in HGPS pathology, we further assessed its localization within the colonic tissue (Catarinella et al. 2022; Ghosh and Vaughan 2012). PAI‐1^+^ cells were significantly increased in the mucosa of mock‐treated *Lmna* ^*G609G/G609G*^ mice compared to *Lmna* ^*+/+*^ controls, consistent with histological evidence of early fibrotic remodeling (Figure 3E,F). While FTI alone had no effect, both Bar and the Bar + FTI therapies significantly reduced PAI‐1^+^ cell abundance (Figure 3E,F). These findings indicate that combination therapy with Bar + FTI alleviates early‐stage mucosal fibrosis in the colon of progeroid mice.

### 2.4 Bar Monotherapy or Combination With FTI Reduces Cellular Senescence in the Colon of HGPS Mice

**FIGURE 4:**
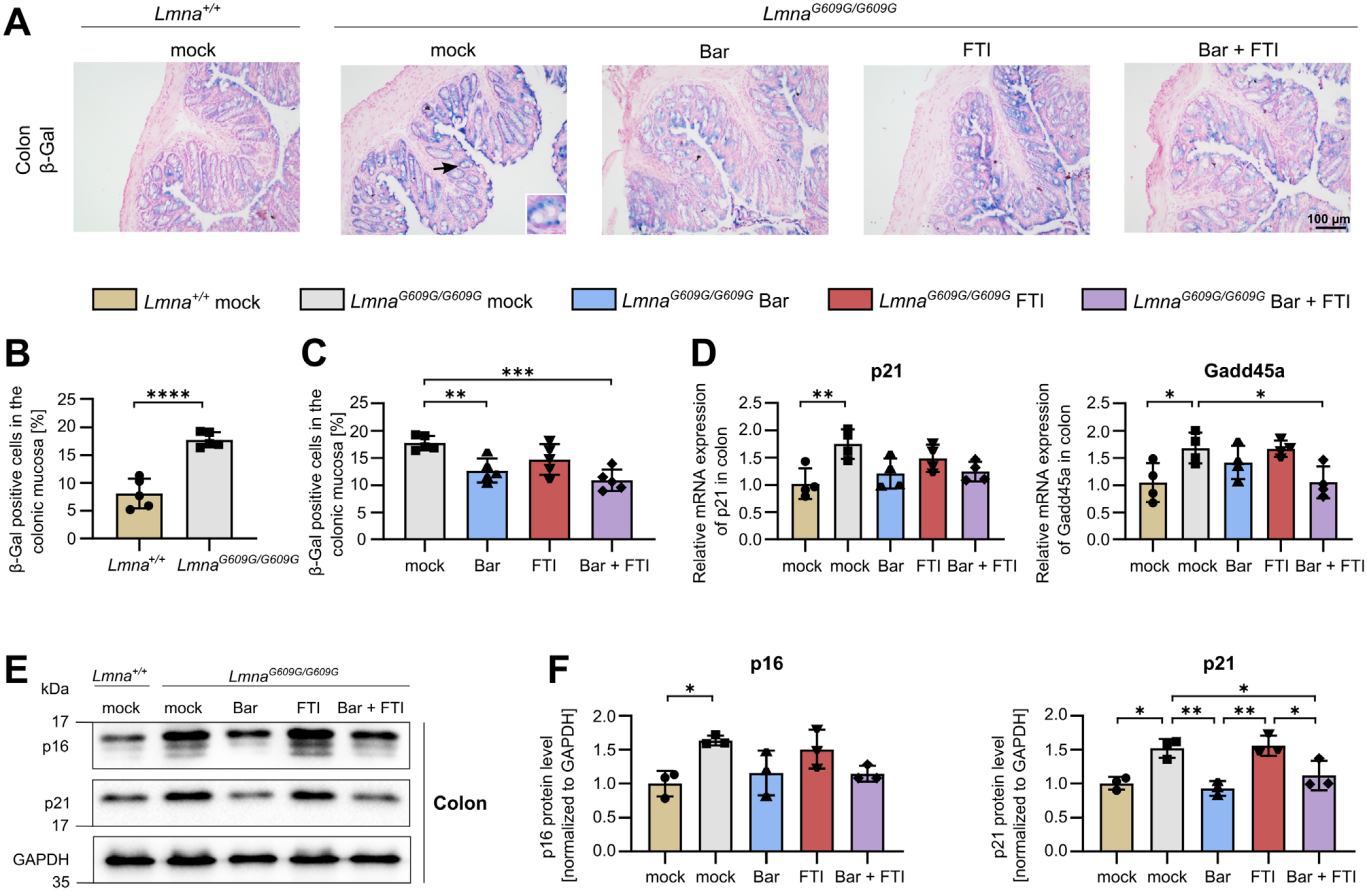
Bar and Bar + FTI treatments attenuate colonic senescence in HGPS mice. (A) Representative β‐galactosidase (β‐Gal) stained images of colon tissue sections from *Lmna* ^*+/+*^ mock, *Lmna* ^*G609G/G609G*^ mock, Bar, FTI or Bar + FTI treated mice. A zoom‐in box is shown for one representative group to illustrate detailed β‐Gal staining. Black arrow indicates the location of the zoomed region. (B) β‐Gal positive cells in the colonic mucosa in *Lmna* ^*+/+*^ mock and *Lmna* ^*G609G/G609G*^ mock mice (*n* = 5). (C) β‐Gal positive cells in the colonic mucosa in treated *Lmna* ^*G609G/G609G*^ mice (*n* = 5). (D) Analysis of mRNA levels of p21 and Gadd45a in the colon of *Lmna* ^*+/+*^ mock, *Lmna* ^*G609G/G609G*^ mock, Bar, FTI, or Bar + FTI treated mice (*n* = 4). (E) WB images of p16 and p21 protein expression in colonic tissue from *Lmna* ^*+/+*^ mock, *Lmna* ^*G609G/G609G*^ mock, Bar, FTI or Bar + FTI treated mice. GAPDH served as a loading control. (F) Densitometric quantification of p16 and p21 based on WB data (normalized to GAPDH expression; *n* = 3). Data are expressed as the mean ± SD. *p < 0.05; **p < 0.01; ***p < 0.001; ****p < 0.0001 calculated using ordinary one‐way ANOVA followed by Tukey's Post Hoc test in *C*, *D*, *and F* and an unpaired *t*‐test in *B*.

Premature cellular senescence contributes to tissue dysfunction and accelerated aging in *Lmna* ^*G609G/G609G*^ progeria mice (Kruger et al. 2024; Osorio et al. 2011). Senescent cells secrete pro‐inflammatory and pro‐fibrotic factors collectively known as the senescence‐associated secretory phenotype, which can exacerbate chronic inflammation, impair tissue regeneration, and promote fibrosis (Saito et al. 2024). Given the elevated inflammatory cytokine levels, impaired epithelial renewal, and increased expression of PAI‐1, which plays a central role in cellular senescence and fibrosis, we next investigated whether these changes were associated with increased senescence in the colon (Vaughan et al. 2017). β‐Galactosidase (β‐Gal) staining revealed a significant increase in β‐Gal^+^ cells in the colonic mucosa of mock‐treated *Lmna* ^*G609G/G609G*^ mice compared to wildtype mice (Figure 4A,B). Positive cells were mainly localized to the surface epithelium and crypt epithelium, with occasional staining in the lamina propria. This accumulation of senescent cells within the mucosa may create a pro‐inflammatory microenvironment that reinforces tissue dysfunction (Tripathi et al. 2021). FTI led to a modest reduction in β‐Gal^+^ cells, whereas Bar alone and Bar + FTI combination therapy produced a more substantial decrease, indicating that JAK–STAT inhibition is particularly effective in suppressing colonic senescence (Figure 4A,C). To further assess the senescent phenotype, we measured the expression of established senescence markers. p21 mRNA was strongly upregulated in mock‐treated L*mna* ^*G609G/G609G*^ mice, with a non‐significant reduction following treatment (Figure 4D). Gadd45a, another p53‐responsive gene implicated in stress‐induced senescence, was also markedly elevated in mock‐treated *Lmna* ^*G609G/G609G*^ mice and notably reduced by Bar + FTI treatment (Figure 4D). Western blot analysis further confirmed increased protein expression of p16 and p21 in mock‐treated *Lmna* ^*G609G/G609G*^ mice (Figure 4E,F). Bar treatment significantly lowered p21 and modestly reduced p16 expression, an effect mirrored by Bar + FTI (Figure 4F), whereas FTI alone had no measurable impact (Figure 4E,F). These findings provide the first evidence that colonic cellular senescence is elevated in HGPS and that JAK–STAT inhibition, either alone or in combination with FTI, effectively reduces senescence in this tissue.

### 2.5 Combination Treatment Preserves Microbial Balance in HGPS Mice Compared to Bar or FTI Single Treatments

**FIGURE 5:**
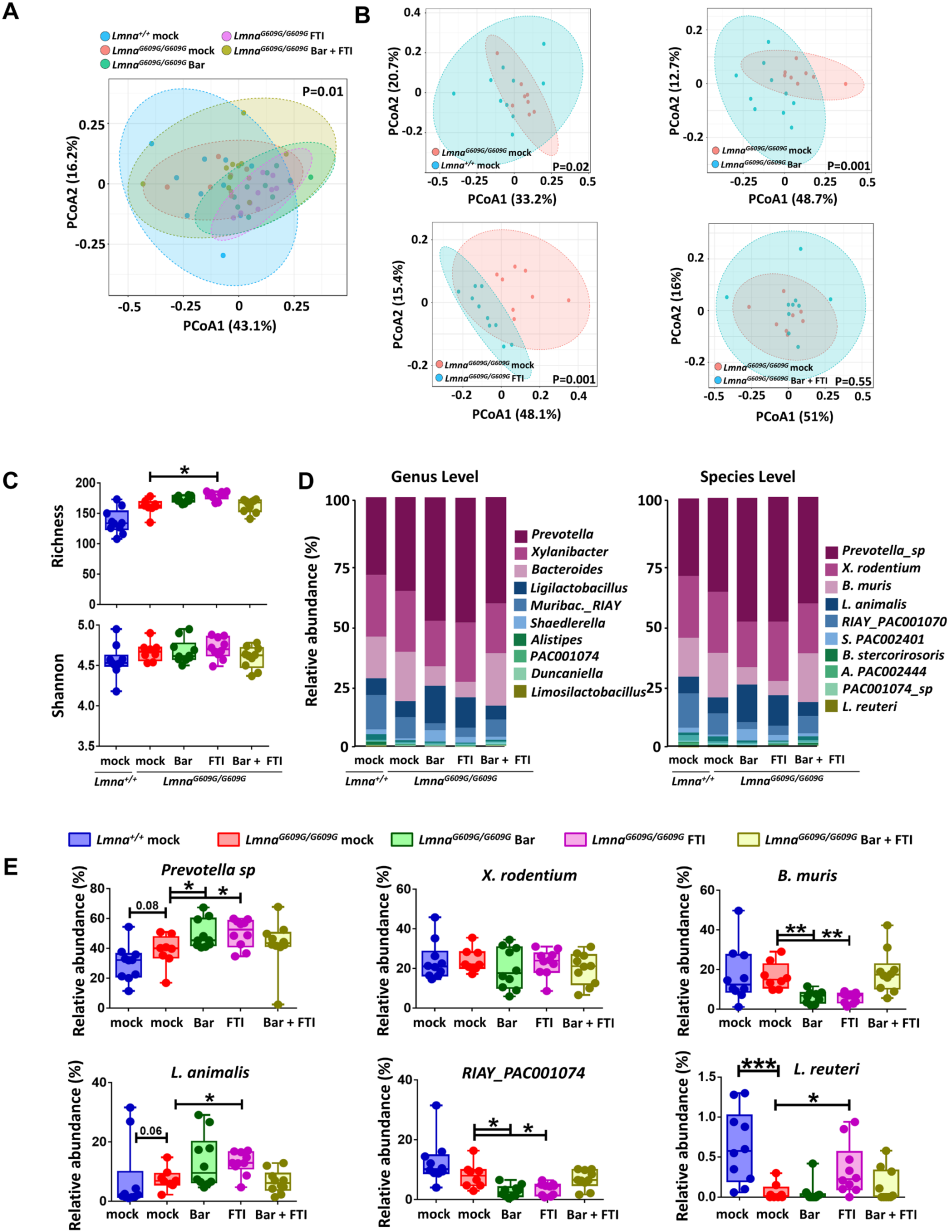
Combination treatment of Bar + FTI preserves microbial balance in HGPS mice more effectively than monotherapies. (A) Principal coordinate analysis (PcoA) plot of β‐diversity of gut microbiota from *Lmna* ^*+/+*^ mock, *Lmna* ^*G609G/G609G*^ mock, and *Lmna* ^*G609G/G609G*^ mice treated with Bar, FTI, or Bar + FTI. Bray–Curtis dissimilarity values were used to calculate the similarity between samples, and statistical significance was tested using PERMANOVA based on the distance matrix. (B) Pairwise PCoA plots comparing *Lmna* ^*G609G/G609G*^ mock mice with *Lmna* ^*+/+*^ mock or *Lmna* ^*G609G/G609G*^ mice treated with Bar, FTI, or Bar + FTI. (C) α‐diversity measures, including species richness (number of observed OTUs) and the Shannon index, across all groups. (D) Bar chart of taxonomy binning displayed at genus and species level. OTU relative abundances were summed up based on shared taxonomic assignments. Taxonomic classification was performed using the Bayesian classifier from the RDP database. (E) Relative abundances of dominant taxa *Prevotella* sp., *X*. *rodentium*, *B. muris* , *L. animalis* , RIAY_PAC001074, and *L. reuteri* , displayed at species level in *Lmna* ^*+/+*^ mock and *Lmna* ^*G609G/G609G*^ mock, Bar, FTI, or Bar + FTI. Each dot represents one mouse. Sample sizes: *n* = 10 for *Lmna* ^*+/+*^ mock and *Lmna* ^*G609G/G609G*^ treated with Bar, FTI, or Bar + FTI; *n* = 8 for *Lmna* ^*G609G/G609G*^ mock mice. All mice were 90 days old at the time of sampling. Statistical significance was assessed using the Kruskal‐Wallis test for panels *C* and *E*. Multiple testing corrections were applied using the Benjamini‐Hochberg procedure. Asterisks indicate statistically significant differences: *p < 0.05, **p < 0.01, ***p < 0.001.

Prior studies in HGPS mouse models have reported gut microbiota shifts that contribute to disease progression, but the impact of therapeutic interventions on the microbiome remains unexplored (Barcena et al. 2019). Based on these findings, we examined how Bar, FTI, and their combination affect gut microbial composition in *Lmna* ^*G609G/G609G*^ mice. To evaluate gut microbiota differences, we analyzed β‐diversity using Principal Coordinates Analysis (PCoA) based on Bray–Curtis dissimilarity. PERMANOVA revealed a significant difference among all five groups, highlighting variation in gut microbiota composition across the studied cohorts (Figure 5A). A separate two‐group comparison confirmed distinct microbiota profiles between *Lmna* ^*+/+*^ and mock‐treated *Lmna* ^*G609G/G609G*^ mice, consistent with genotype‐driven dysbiosis in progeria (Figure 5B). Pairwise comparisons of each treated group with mock‐treated *Lmna* ^*G609G/G609G*^ mice showed that combined treatment induced a weaker shift in microbiome composition than the single treatments (Figure 5B). Despite differences in β‐diversity, α‐diversity, as assessed by species richness and Shannon index, remained largely unchanged across groups at 90 days of age, indicating that overall diversity within individual samples was preserved (Figure 5C). Interestingly, only FTI treatment significantly increased species richness, while the Shannon index remained stable, indicating minor community evenness and limited contributions from low‐abundance new taxa (Figure 5C). Next, we examined the relative abundance of gut microbial taxa to identify bacteria enriched in progeroid mice and assess whether their levels were affected by Bar, FTI, or combination treatment. At the phylum level, no major differences were observed between combination‐treated and mock‐treated *Lmna* ^*G609G/G609G*^ mice, whereas Bar and FTI monotherapies increased the relative abundance of Firmicutes compared to healthy *Lmna* ^*+/+*^ controls (Figure S7A). This trend was consistent at the genus level, where Bar‐ and FTI‐treated mice clustered closely, suggesting similar effects on microbiota composition (Figure 5D, Figure S7B). At the species level, *Prevotella* sp. exhibited a moderate increase in relative abundance in mock‐treated *Lmna* ^*G609G/G609G*^ mice compared to wildtype and was further enriched following Bar and FTI monotherapies (Figure 5D,E, Figure S7C). Other taxa, such as *Ligilactobacillus animalis*, *Bacteroides muris*, and RIAY_PAC001074, were primarily altered by Bar and FTI monotherapies, while their relative abundances in the combination‐treated group remained more balanced and aligned more closely with wildtype levels. Although *Limosilactobacillus reuteri* was a low‐abundance taxon overall, its relative abundance was further reduced in mock‐treated *Lmna* ^*G609G/G609G*^ mice and showed an increase under FTI treatment (Figure 5D,E). Predictive functional pathway analysis (PICRUSt) suggests that Bar and FTI monotherapies may enhance the biosynthesis of essential amino acids, as reflected by the upregulation of the aromatic amino acid and chorismate biosynthesis pathways (Figure S8A,B). Additionally, the N‐acetylglucosamine degradation pathway was enriched upon both treatments. Conversely, mock *Lmna* ^*G609G/G609G*^ microbiota exhibited a clear increase in L‐isoleucine biosynthesis pathway (Figure S8A–C). Interestingly, with the treatment combination, only a few pathways were altered, displaying a drastic decrease in the anhydromuropeptides recycling and aerobic respiration pathways (Figure S8C). While β‐diversity analyses confirmed differences between wildtype and mock‐treated progeroid mice, the extent of dysbiosis at this time point was relatively modest. Bar and FTI monotherapies induced similar compositional changes, whereas combination treatment appeared to stabilize the microbiota, preserving a profile closer to that of healthy controls. In contrast, progeroid mice at a later disease stage (114–122 days old) showed more severe microbiome disruption, with greater divergence in β‐diversity from wildtype and a significant decline in Shannon index values (Figure S9A,B). Notably, *Prevotella* sp. showed a marked increase in relative abundance at this late stage (Figure S9C). At the same time, *Lactobacillus johnsonii* , a commonly reported commensal species, was significantly depleted, suggesting that gut microbiota composition becomes progressively more disrupted as HGPS advances (Figure S9C) (Zhang, Zhao, et al. 2023).

## 3 Discussion

Our findings demonstrate that the JAK–STAT inhibitor Bar combined with FTI effectively alleviates intestinal dysfunctions and inflammation in the *Lmna* ^*G609G/G609G*^ mouse model. While JAK–STAT inhibition has been linked to systemic benefits in HGPS, our findings highlight a critical role in colonic homeostasis, a previously underexplored aspect of progeria. Although FTI provides clear clinical benefits, its associated cellular toxicities and GI side effects underscore the need for adjunctive therapies (Arnold et al. 2021; Eiger BioPharmaceuticals Inc. 2020; Kamasani et al. 2004; Krüger et al. 2025; Li et al. 2019; Muela‐Zarzuela et al. 2024; Verstraeten et al. 2011).

Emerging preclinical data suggest that GI dysfunctions may contribute to disease progression. A high‐fat diet in *Lmna* ^*G609G/G609G*^ extended survival but exacerbated disease phenotypes, including intestinal defects. In addition, histopathological alterations have been reported throughout the GI tract (Barcena et al. 2018; Hou et al. 2023; Kreienkamp et al. 2019; Wang et al. 2024; Zhang, Hu, et al. 2023). Gut microbiota imbalance has been linked to systemic decline, while fecal microbiota transfer improved lifespan and healthspan (Barcena et al. 2019). In line with these studies, we observed the presence of microbiome alterations in progeroid mice and showed that dysbiosis becomes more pronounced at a later disease stage. This includes an increase in an unidentified species of *Prevotella*, a genus particularly noteworthy for its link to mucosal inflammation (Iljazovic et al. 2021; Larsen 2017). Conversely, the relative abundance of *Lactobacillus johnsonii* , a probiotic strain known for its epithelial‐protective and anti‐inflammatory properties, was markedly reduced at late stages of the disease (Tao et al. 2025; Zhang, Zhao, et al. 2023). Taxonomic analysis revealed a distinct microbial profile in stool samples of both wild‐type and progeroid mice, predominantly composed of the Bacteroidetes and Firmicutes phyla. Notably, members of the Proteobacteria and Verrucomicrobia were not detected. We hypothesize that this restricted distribution may be influenced by pre‐analytical and environmental factors, such as sample handling, animal facility conditions, and diet. The microbial changes were accompanied by reduced crypt depth, thinning of the muscularis mucosa, goblet cell loss, impaired epithelial regeneration markers, and increased senescent markers. At the molecular level, we observed chronic activation of the JAK–STAT and NF‐κB pathways and increased expression of pro‐inflammatory cytokines. We also observed fibrotic changes in the mucosa, including modest but significant increases in collagen deposition and PAI‐1^+^ cell abundance. These changes may represent the initial stages of fibrosis that could advance with age, especially given the chronic and progressive nature of HGPS. In IBD mouse models, fibrosis progresses over time, with collagen accumulation increasing as the disease advances (Steiner et al. 2023; Suzuki et al. 2011).

In our model, FTI treatment aggravated inflammatory signaling, resulting in pronounced STAT1 hyperactivation and greater upregulation of cytokine and chemokine gene expression compared to mock‐treated progeroid mice. These in vivo findings are consistent with in vitro reports demonstrating FTI‐induced inflammation through activation of the cGAS‐STING‐STAT1 axis, as well as elevated IL‐6 secretion following FTI exposure (Arnold et al. 2021; Li et al. 2019). In contrast, Bar effectively suppressed STAT1 and STAT3 hyperactivation and reduced NF‐κB activation. Notably, the addition of Bar to FTI not only reduced the baseline inflammatory phenotype but also prevented FTI‐induced exacerbation of inflammation. Beyond inflammation, Bar and Bar + FTI reduced markers of cellular senescence in the colon, whereas FTI alone had no effect. Importantly, only the combination therapy was effective in reversing early signs of colonic fibrosis. These results align with a previous report demonstrating that Bar + FTI reduced dermal and vascular fibrosis, an effect not observed with FTI alone (Krüger et al. 2025). Bar + FTI therapy also preserved gut microbiota composition more similar to the wildtype mice profile. While Bar and FTI monotherapies induced distinct microbiota shifts, their combination appeared to buffer against these alterations. Predictive functional pathway analysis suggested that Bar and FTI monotherapies enhanced amino acid biosynthesis, indicating a shift toward beneficial metabolite production, but also enriched N‐acetylglucosamine degradation, which could compromise mucin‐dependent barrier integrity (Neis et al. 2015; Raimondi et al. 2021). The microbiota of mock‐treated *Lmna* ^*G609G/G609G*^ mice showed increased L‐isoleucine production, an essential amino acid for barrier integrity, suggesting that despite dysbiosis, part of the microbiota may preserve a beneficial role in maintaining intestinal function. Combination therapy regulated only a few pathways, consistent with its milder microbial shifts. Reduction in the anhydromuropeptides recycling pathway suggests more stable bacterial growth with a reduced metabolism of bacterial cell walls (Johnson et al. 2013). Enrichment of aerobic respiration pathways may reflect a shift of the microbial community toward more oxygen‐tolerant species or that there is an increased availability of oxygen in the gut lumen. Importantly, an increase in oxygen in the gut can be a sign of inflammation (Rigottier‐Gois 2013; Zeng et al. 2017). These findings also suggest that gut microbial composition is influenced not only by inflammation but also by broader aspects of intestinal homeostasis, which was best preserved by the Bar + FTI combination. This observed therapeutic benefit of the combination likely reflects complementary actions. FTI inhibits progerin farnesylation, while Bar acts on downstream inflammatory pathways triggered by progerin accumulation (Bidault et al. 2020; Capell et al. 2005). This is further supported by our previous study, where the same treatment regimen led to greater lifespan extension than either monotherapy (Krüger et al. 2025).

Our results further suggest that combining Bar with FTI may not only improve GI health in progeroid mice but may also help mitigate GI side effects frequently associated with FTI. In the clinical trial leading to FTI's approval, GI symptoms were the most frequently reported adverse events, with vomiting (93%), diarrhea (84%), nausea (56%), and appetite loss (53%) being particularly common (Eiger BioPharmaceuticals Inc. 2020). Nearly half of the patients who experienced vomiting required medical intervention, and several continued to rely on anti‐emetics or loperamide for symptom management (Eiger BioPharmaceuticals Inc. 2020). This is especially concerning in HGPS, as affected children often rely on small, high‐calorie meals that are ideally consumed frequently throughout the day (Progeria Research Foundation 2022). In this context, our findings support combining Bar with FTI, an interpretation strengthened by Bar's benefits in other inflammatory gut conditions. In a pilot study of refractory intestinal Behçet's disease, Bar induced mucosal healing with 77% complete remission (Liu et al. 2023). Bar has also shown efficacy in preclinical ulcerative colitis, and other JAK–STAT inhibitors are already approved for treating IBDs (Honap et al. 2024; Wu et al. 2024). Bar's established clinical profile further strengthens its translational potential for HGPS. It is approved for rheumatoid arthritis, alopecia areata, and atopic dermatitis and is licensed for pediatric use (≥ 2 years) in juvenile idiopathic arthritis and atopic dermatitis (European Medicines Agency 2023; Ramanan et al. 2023; Torrelo et al. 2023). Its once‐daily oral dosing provides a practical advantage for chronic pediatric care, and long‐term safety data in rheumatoid arthritis (up to 9.3 years) revealed no new safety signals (Taylor et al. 2022).

Beyond HGPS, JAK–STAT inhibition may also benefit the normal aging colon. Aging is characterized by chronic, low‐grade inflammation (inflammaging), which contributes to dysbiosis and impaired barrier function (Lopez‐Otin et al. 2023; Zhang, Yan, et al. 2023). Interestingly, JAK–STAT inhibition reduced systemic inflammation and improved physical function in aged mice (Xu et al. 2015). Whether these benefits extend to the physiological colonic aging remains to be tested. In addition, the known adverse effects of these agents carry risks that are particularly relevant in older adults, including serious infections, malignancies, and thrombosis and therefore require clinical monitoring (Eli Lilly and Company 2022).

This study was conducted as part of a broader investigation into lifespan, healthspan, and cardiovascular outcomes in HGPS mice (Krüger et al. 2025). While our study focused on histological and molecular colon features, future investigations should include functional assays assessing gut motility, permeability, nutrient absorption, and metabolomics to fully elucidate the physiological impact of Bar + FTI therapy. Approaches such as organoid culture or in vivo proliferation assays will be important to directly test whether Bar + FTI restores epithelial regeneration, whereas single‐cell RNA sequencing could define the specific cellular drivers of inflammation and senescence in the HGPS colon. While we assessed colonic pathology and observed fibrosis, inflammation, senescence, impaired epithelial renewal, and microbial dysbiosis occurring in parallel, further mechanistic work will be critical to determine whether and how these processes are causally linked. Together, these findings deepen our understanding of GI pathology in HGPS and support Bar + FTI combination therapy as a promising means to enhance treatment efficacy while minimizing side effects. With FTI as the standard of care, Bar offers a well‐tolerated and complementary adjunct. Clinical studies will be essential to validate its utility in HGPS and other age‐related diseases.

## 4 Material and Methods

### 4.1 Mouse Model

C57BL/6 mice carrying the *Lmna* ^*G609G*^ mutation were kindly provided by Prof. Carlos‐Lopes Otin (University of Oviedo, Spain) and have been previously described (Osorio et al. 2011; Zaghini et al. 2020). All animal procedures were approved by the Bavarian state government and conducted in accordance with the Animal Welfare Act. To establish a specific‐pathogen‐free (SPF) status, embryo transfer was used at colony initiation. Mice were maintained under strict SPF‐grade pathogen‐free conditions throughout the study in individually ventilated cages (IVCs). The housing facility was set on a 12‐h light/dark cycle at a controlled temperature of 21°C–22°C with 50% relative humidity. Animals were separated by sex (maximum five per cage) and had ad libitum access to food and water. Bedding and drinking water were autoclaved. All chows, including treatment and control diets, were irradiated at 25 kGy to ensure sterility. This study used mice from a previously published treatment experiment (Krüger et al. 2025). A total of 100 mice were included in this experiment. At postnatal Day 28, animals were randomly assigned to one of five groups (*n* = 20 per group): *Lmna* ^*+/+*^ mock, *Lmna* ^*G609G/G609G*^ mock, *Lmna* ^*G609G/G609G*^ Baricitinib (Bar), *Lmna* ^*G609G/G609G*^ Lonafarnib (FTI), and *Lmna* ^*G609G/G609G*^ Bar + FTI. Treatments began immediately following group allocation. Mock‐treated mice received standard chow (PS RM‐H, V1534; ssniff Spezialdiäten GmbH). Treatment groups were fed standard chow supplemented with Bar (62.5 mg/kg diet, MedChemExpress; HY‐15315), FTI (187.5 mg/kg diet; kindly provided by the PRF), or both. All compounds were incorporated into the chow during commercial preparation by ssniff Spezialdiäten GmbH to ensure homogenous distribution. Dosages refer to milligrams of compound per kilogram of diet. From 8 weeks of age onward, all mice were provided with water‐soaked chow to facilitate consumption. Mice were monitored for health status and body weight, as previously detailed (Krüger et al. 2025). Mice were sacrificed at 90 days of age. For Figure S9, microbiome analysis was performed on mice nearing their humane endpoint, between 114 and 122 days of age. Tissue samples for histological and molecular analyses were collected from the distal colon.

### 4.2 Genotyping

DNA was extracted using earmark punches collected at weaning. After incubating the tissue in 50 mM NaOH at 95°C for 30 min, 1 M Tris buffer (pH = 8.0) was added for neutralization. PCR was performed using published primers (Osorio et al. 2011) according to the amplification protocol described previously (Zaghini et al. 2020). PCR products were analyzed by agarose gel electrophoresis.

### 4.3 Western Blot

Mouse colonic tissues were snap‐frozen in liquid nitrogen and homogenized in RIPA buffer containing PMSF (Cell Signaling, 8553), protease (Thermo Fisher, 78,430), and phosphatase inhibitors (Thermo Fisher, 78,426). Lysates were centrifuged at 13,000×*g* for 15 min at 4°C, and protein concentrations were determined by Bradford assay (BioRad, 5,000,206). Equal amounts of protein were separated on SDS‐PAGE (8%–15%) and transferred to nitrocellulose membranes. Membranes were blocked with 5% milk and incubated overnight at 4°C with primary antibodies (see Table 1). After TBS‐T washes, membranes were incubated with HRP‐conjugated anti‐rabbit or anti‐mouse secondary antibodies (1:5000, Jackson ImmunoResearch Laboratories). Detection was performed using ECL substrate (Bio‐Rad) and visualized with a ChemiDoc MP system. Band intensities were quantified using ImageLab and normalized to GAPDH. Membranes were stripped using a stripping buffer (Thermo Fisher, 46,430) and reprobed as needed.

**TABLE 1:** Primary antibodies for immunofluorescence and western blot.

Antibody	Isotype	Company	Dilution	Application	Cat. no
IRF1	Rabbit mAB	Cell signaling	1:200	Immunofluorescence	#8478
Ki67	Rabbit pAB	Abcam	1:800	Immunofluorescence	ab15580
Lamin A	Rabbit pAB	Sigma‐Aldrich	1:800	Immunofluorescence	L1293
LGR5	Mouse mAB	Invitrogen	1:500	Immunofluorescence	OTI2A2
PAI‐1	Mouse mAB	Invitrogen	1:500	Immunofluorescence	MA5‐17171
Vimentin	Rabbit mAB	Cell signaling	1:1000	Immunofluorescence	#5741
Vimentin (maB7A3)	Mouse mAB	(Papamarcaki et al. 1991)	1:50	Immunofluorescence	N/A
α‐smooth muscle actin	Mouse mAB	Sigma‐Aldrich	1:1000	Immunofluorescence	C6198
GAPDH	Rabbit pAB	Sigma‐Aldrich	1:10000	Western blot	G9545
Lamin A	Rabbit pAB	Sigma‐Aldrich	1:2000	Western blot	L1293
Lamin A/C	Rabbit pAB	Santa Cruz	1:5000	Western blot	sc20681
NfκB p65	Rabbit mAB	Cell signaling	1:1000	Western blot	#8242
p16 INK4a	Mouse mAB	Invitrogen	1:1000	Western blot	MA5‐17142
p21 Waf1/Cip1	Mouse mAB	Santa Cruz	1:250	Western blot	sc‐6246
P‐NfκB p65	Rabbit mAB	Cell signaling	1:1000	Western blot	#3033
P‐STAT1	Rabbit mAB	Cell signaling	1:800	Western blot	#9167
P‐STAT3	Rabbit mAB	Cell signaling	1:1000	Western blot	#9145
STAT1	Rabbit mAB	Cell signaling	1:1000	Western blot	#14994
STAT3	Mouse mAB	Cell signaling	1:1000	Western blot	#9139

### 4.4 Immunofluorescence

Colon tissues were sectioned at 6 μm and stored at −80°C until use. Sections were fixed either in 4% PFA (Merck, 104,005) for 10 min at room temperature or in methanol at −20°C for 10 min, washed with PBS and permeabilized with 0.2% Triton X‐100 in PBS for 10 min. After washing, samples were blocked for 1 h in 15% FBS (Gibco, 10,270,106) and incubated overnight at 4°C with primary antibodies (see Table 1). Alexa Fluor 488‐conjugated anti‐rabbit (1:1000, Invitrogen, A21206) and anti‐mouse secondary antibodies (1:1000, Invitrogen, A31570) were applied, followed by DAPI counterstaining using Vectashield mounting medium (Vector, H‐1200). Images were acquired on a Keyence BZ‐X810 fluorescence microscope under fixed exposure settings for each marker to ensure consistency across samples. Quantification was performed in ImageJ (NIH, version 1.54f). Ki67, Lgr5, and PAI‐1‐positive cells were quantified relative to DAPI‐positive nuclei, while vimentin was expressed as percentage positive area. Detailed image acquisition and quantification procedures are provided in the Supporting Information under Materials and Methods.

### 4.5 Quantitative Reverse Transcription PCR

Total RNA was extracted from colonic tissue using the Gene JET RNA Purification Kit (Thermo Fisher, K0731) according to the manufacturer's instructions. RNA yield and purity were assessed using a NanoDrop spectrophotometer (Thermo Fisher). For cDNA synthesis, 800 ng of RNA was reverse transcribed using the High‐Capacity cDNA Reverse Transcription Kit (Thermo Fisher). PCR was performed on a StepOnePlus Real‐Time PCR System (Thermo Fisher) with PowerUp SYBR Green Master Mix (Applied Biosystems). Each 10 μL reaction included 1 μL cDNA (4 ng), 5 μL master mix, 2 μL RNase‐free water, and 1 μL of each primer (300 nm, forward/reverse). Cycling conditions were: 50°C for 2 min, 95 C for 10 min, followed by 45 cycles of 95°C for 15 s and 60°C for 1 min. Relative expression was calculated using the ΔΔCT method (Fleige et al. 2006), with GAPDH as the endogenous control. All reactions were run in triplicates using four biological replicates. Primers are listed in Table 2.

**TABLE 2:** Primer sequences used for qPCR.

Target gene	GenID	Forward primer sequence 5′	Reverse primer sequence 5′
Ccl2	20,296	TGTGAGTTACATACCCCGGC	GCCTGAACAGCAGCCATAGA
Claudin‐1	12,737	GGACTGTGGATGTCCTGCGTTT	GCCAATTACCATCAAGGCTCGG
Cxcl1	14,825	CTGGGATTCACCTCAAGAACATC	CAGGGTCAAGGCAAGCCTC
Cxcl10	15,945	CCAAGTGCTGCCGTCATTTTC	GGCTCGCAGGGATGATTTCAA
Cxcl5	20,311	TGCGTTGTGTTTGCTTAACCGTAAC	TGACTTCCACCGTAGGGCACTG
F11r	16,456	CACCTACTCTGGCTTCTCCTCT	TGCCACTGGATGAGAAGGTGAC
Gadd45a	13,197	CCTGGAGGAAGTGCTCAGCAAG	GTCGTCTTCGTCAGCAGCCAG
Gapdh	14,433	AGGTCGGTGTGAACGGATTTG	TGTAGACCATGTAGTTGAGGTCA
Il‐1a	16,175	GCACCTTACACCTACCAGAGT	AAACTTCTGCCTGACGAGCTT
Il‐1b	16,176	TGGACCTTCCAGGATGAGGACA	GTTCATCTCGGAGCCTGTAGTG
Il‐6	16,193	TAGTCCTTCCTACCCCAATTTCC	TTGGTCCTTAGCCACTCCTTC
Ki67	17,345	GAGGAGAAACGCCAACCAAGAG	TTTGTCCTCGGTGGCGTTATCC
Lgr5	14,160	AGAGCCTGATACCATCTGCAAAC	TGAAGGTCGTCCACACTGTTGC
Occludin	18,260	TGGCAAGCGATCATACCCAGAG	CTGCCTGAAGTCATCCACACTC
p21	12,575	TCGCTGTCTTGCACTCTGGTGT	CCAATCTGCGCTTGGAGTGATAG
Pai1	18,787	TTCAGCCCTTGCTTGCCTC	ACACTTTTACTCCGAAGTCGGT
Tgfb1	21,803	CTCCCGTGGCTTCTAGTGC	GCCTTAGTTTGGACAGGATCTG
Tnf‐α	21,926	CCTGTAGCCCACGTCGTAG	GGGAGTAGACAAGGTACAACCC
Zo‐1	21,872	GTTGGTACGGTGCCCTGAAAGA	GCTGACAGGTAGGACAGACGAT

### 4.6 High‐Throughput 16S‐Ribosomal RNA (rRNA) Gene Amplicon Sequencing Analysis

Fecal samples were collected from the colon during necropsy and immediately snap‐frozen in liquid nitrogen. 16S rRNA gene amplicon sequencing was conducted at the ZIEL—Core Facility Microbiome of the Technical University of Munich (Reitmeier et al. 2020). DNA extraction was performed as previously described in detail, using the Maxwell RSC Fecal Microbiome DNA Kit in the Maxwell RSC Instrument after bead beating, according to the manufacturer's instructions (Reitmeier et al. 2020). The V3–V4 regions of the 16S rRNA gene were amplified via a two‐step PCR process, purified with magnetic beads and sequenced on an Illumina MiSeq platform (2 × 300 bp), generating up to 25 million 2 × 300 bp reads. Raw sequencing data were analyzed in a UPARSE‐based pipeline using the IMNGS2 platform, an updated version of IMNGS with taxonomy‐informed clustering (Kioukis et al. 2022; Lagkouvardos et al. 2016). Briefly, primer and barcode sequences were trimmed from each read and low‐quality reads, chimeras and sequences shorter than 200 bp were removed (Wang et al. 2007). An average of 3.35 × 10^4^ clean reads were obtained per sample. They were clustered into zero‐radius operational taxonomic units (zOTUs) at 97% sequence similarity and taxonomically classified using the RDP classifier. Noteworthy, we have previously optimized our workflow for sample handling and analysis using mock communities, and our method enabled accurate taxa identification at the species level with up to 99% similarity (Amar et al. 2021). Downstream analysis of zOTU tables was conducted using the Rhea pipeline (Lagkouvardos et al. 2017). Spurious taxa were filtered out using a prevalence cut‐off of 0.25% and an abundance cut‐off of 0.5%, and the obtained read counts were normalized across samples using the total sum scaling method (TSS) (Reitmeier et al. 2021). Based on these cut‐offs, 44 OTUs were removed from a total number of 338 taxa. Alpha‐diversity was assessed using taxa richness and the Shannon index of zOTUs. Beta‐diversity was calculated using Bray–Curtis dissimilarity values and visualized via principal coordinate analysis (PCoA). Statistical significance of group separation in beta‐diversity space was determined using PERMANOVA. Taxonomic differences between groups were assessed using the Kruskal–Wallis rank sum test for multiple group comparisons and the Mann–Whitney test for pairwise comparisons. Multiple testing corrections were performed using the Benjamini–Hochberg procedure, with corrected *p*‐values ≤ 0.05 considered statistically significant.

To infer the functional potential of the gut microbiome from mouse stool samples, the Phylogenetic Investigation of Communities by Reconstruction of Unobserved States (PICRUSt) pipeline was employed (Douglas et al. 2020). The PICRUSt2 tool places amplicon sequence variants (ASVs) onto a comprehensive reference phylogenetic tree of known microbial genomes. Using ancestral state reconstruction, it predicts the presence and abundance of gene families for each ASV based on the gene content of its closest sequenced relatives. These individual predictions are then aggregated to generate a comprehensive functional profile based on the MetaCyc database. The quality of these predictions was assessed using the Nearest Sequenced Taxon Index (NSTI), which provides a score based on the phylogenetic distance of each ASV from a known reference genome. For statistical analysis, the functional data was normalized to correct for differences in sequencing depth, and differential abundance analysis was performed using DESeq2 to identify pathways that were significantly enriched or depleted between treatment and control groups.

### 4.7 Histological Staining

Mice were euthanized by cervical dislocation under 5% isoflurane anesthesia and perfused with 20 mL PBS (Sigma‐Aldrich). Distal colonic tissues were collected, embedded in OCT (Sakura, 4583) using cryo‐molds (Sakura, 4565), and snap‐frozen in liquid nitrogen. Sections (6 μm) were prepared using a Leica CM3050S cryotome and stained with Hematoxylin and Eosin (Abcam, ab245880), Masson's Trichrome (Abcam, ab150686), Periodic Acid‐Schiff (Sigma‐Aldrich, 395B), or β‐Gal. Histological analysis was performed on colonic tissues from five animals per group using ImageJ (NIH, version 1.54f). Quantification was performed under fixed exposure and threshold settings across all samples. Crypt depth, muscularis mucosa thickness, and goblet cell counts were measured from H&E and PAS sections. Collagen‐positive area was quantified from Masson's trichrome images, and β‐Gal‐positive cells were calculated as the percentage of total nuclei. Detailed protocols used for tissue staining and subsequent image analysis are described in the Supporting Information under Materials and Methods.

## Author Contributions

K.D., P.K., and M.S. contributed to the conception of the study and designed the experiments. K.N. performed 16S amplicon sequencing. Y.A. conducted the microbiome data analysis and prepared the corresponding figures. M.S. performed the experiments and prepared the figures. P.K. and M.S. carried out the mouse treatments and sample collections. K.D. and M.S. analyzed the data. K.D., M.S., and Y.A. wrote the manuscript. K.D. supervised the project and acquired funding. All authors have read and agreed to the published version of the manuscript.

## Conflicts of Interest

The authors declare no conflicts of interest.

## Supporting information


**Appendix S1:** acel70273‐sup‐0001‐AppendixS1.docx.

## Data Availability

16S rRNA sequencing data have been deposited in NCBI Submission Platform and are available under the following link: https://submit.ncbi.nlm.nih.gov/subs/sra/SUB15340525/files. All other study data are included in the article and/or SI Appendix.
