# Supplementary material for: Baricitinib Augments Lonafarnib Therapy to Preserve Colonic Homeostasis and Microbial Balance in a Mouse Model of Progeria
Source: Aging Cell. 2025 Oct 20;24(12):e70273. doi: 10.1111/acel.70273 (PMC12686558; doi:10.1111/acel.70273)
Supplement: Supplementary file 1 — Appendix S1: acel70273‐sup‐0001‐AppendixS1.docx. [file ACEL-24-e70273-s001.docx]

**Supporting Information for**

Baricitinib Augments Lonafarnib Therapy to Preserve Colonic Homeostasis and Microbial Balance in a Mouse Model of Progeria

Moritz Schroll^1^, Yacine Amar^2^, Peter Krüger^1^, Klaus Neuhaus^3^, Karima Djabali^1*^

1 Epigenetics of Aging, Department of Dermatology and Allergy, TUM School of Medicine and Health, Munich Institute of Biomedical Engineering, Technical University of Munich, 85748 Garching, Germany.

2 Department of Dermatology and Allergy, School of Medicine, Technical University of Munich, Munich 80802, Germany

3 Core Facility Microbiome, ZIEL - Institute for Food & Health, Technical University of Munich, Weihenstephaner Berg 3, 85354 Freising, Germany

*Karima Djabali

**Email:**  djabali@tum.de

**This PDF file includes:**

Figures S1 to S9

Supporting Information Material and Methods


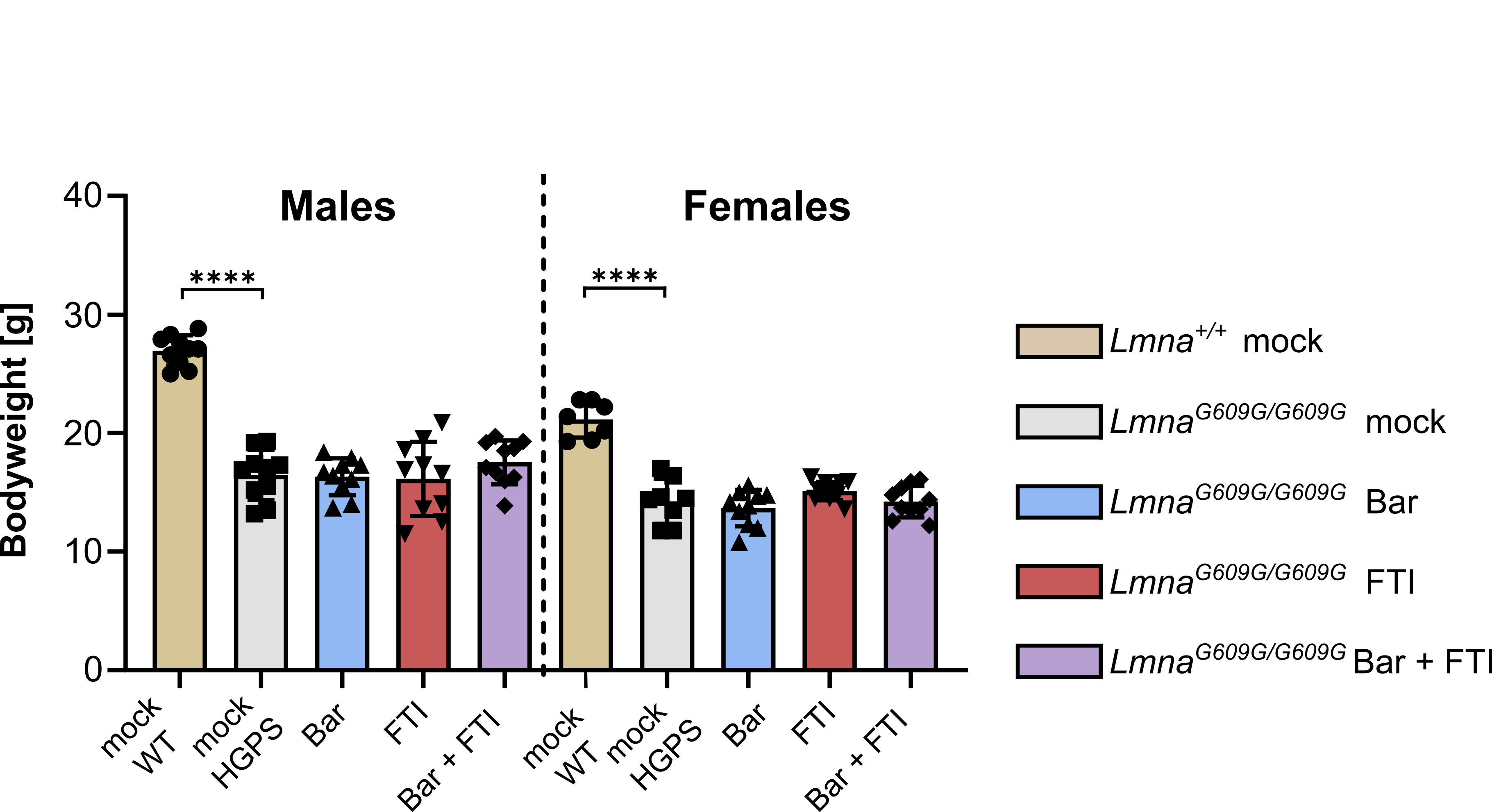


Figure S1. Comparable body weight across progeroid mice. Analysis of bodyweight of male and female *Lmna^+/+^* mock, *Lmna^G609G/G609G^* mock, Bar, FTI and Bar + FTI treated mice at day 90 (n = 5 - 10 per group). Data are expressed as the mean ± SD. ****p < 0.0001, calculated using ordinary one-way ANOVA followed by Tukey´s Post


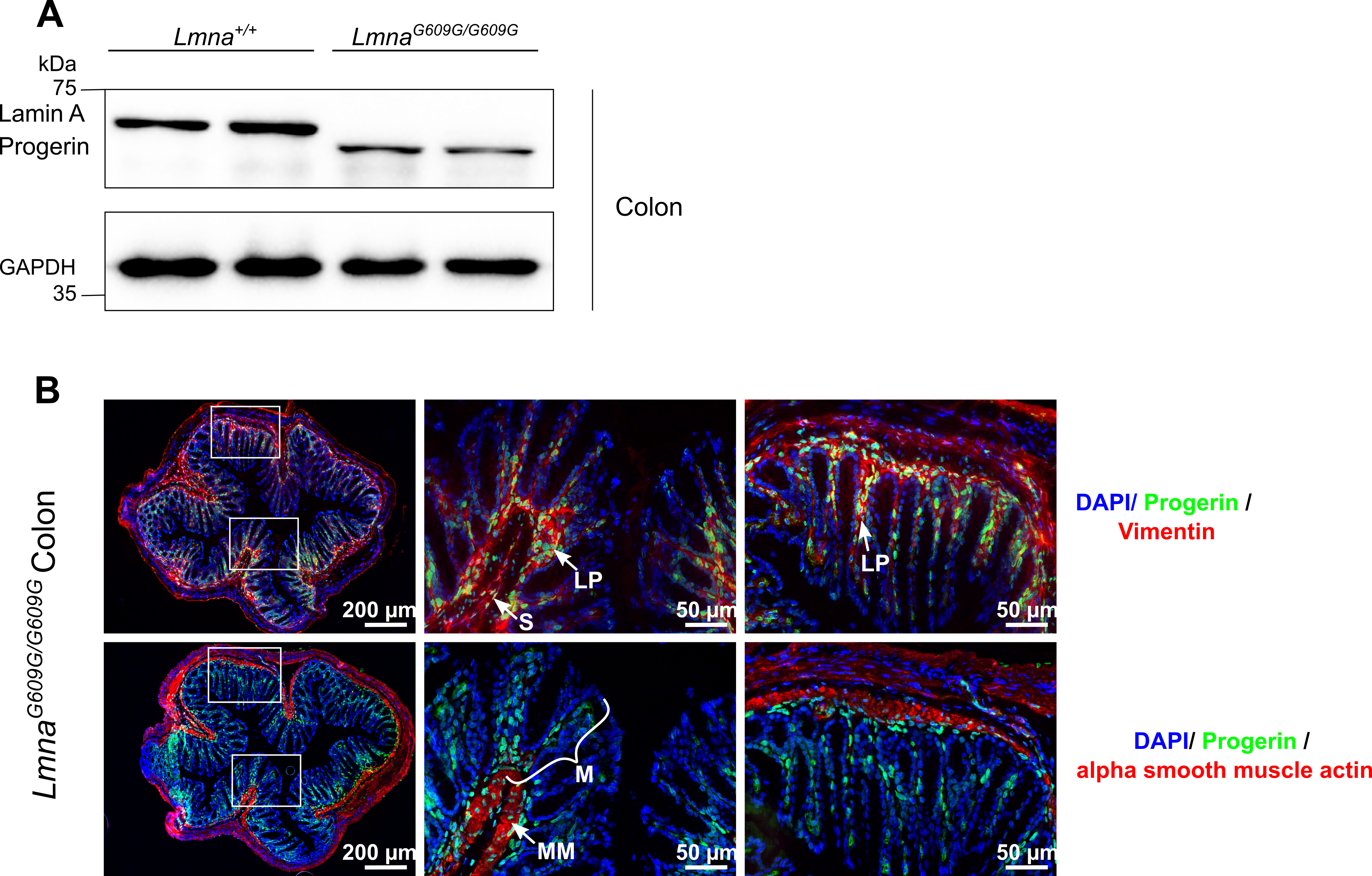
Figure S2. Progerin expression in HGPS mice. (A) WB image of Lamin A/progerin protein expression in colonic tissue from *Lmna^+/+^* and *Lmna^G609G/G609G^* mice validating that the Lamin A antibody predominantly detects progerin in progeroid mice. GAPDH was used as a loading control. (B) Immunofluorescence images of colon tissue from *Lmna^G609G/G609G^* mice. Upper panels: Vimentin (red) and progerin (green). Lower panels: alpha smooth muscle actin (red) and progerin (green). Nuclei were counterstained with DAPI (blue). Images shown at 10× magnification (scale bar, 200 µm) and 40× magnification (scale bar, 50 µm). Abbreviations: lamina propria (LP), mucosa (M), muscularis mucosa (MM), submucosa (S)


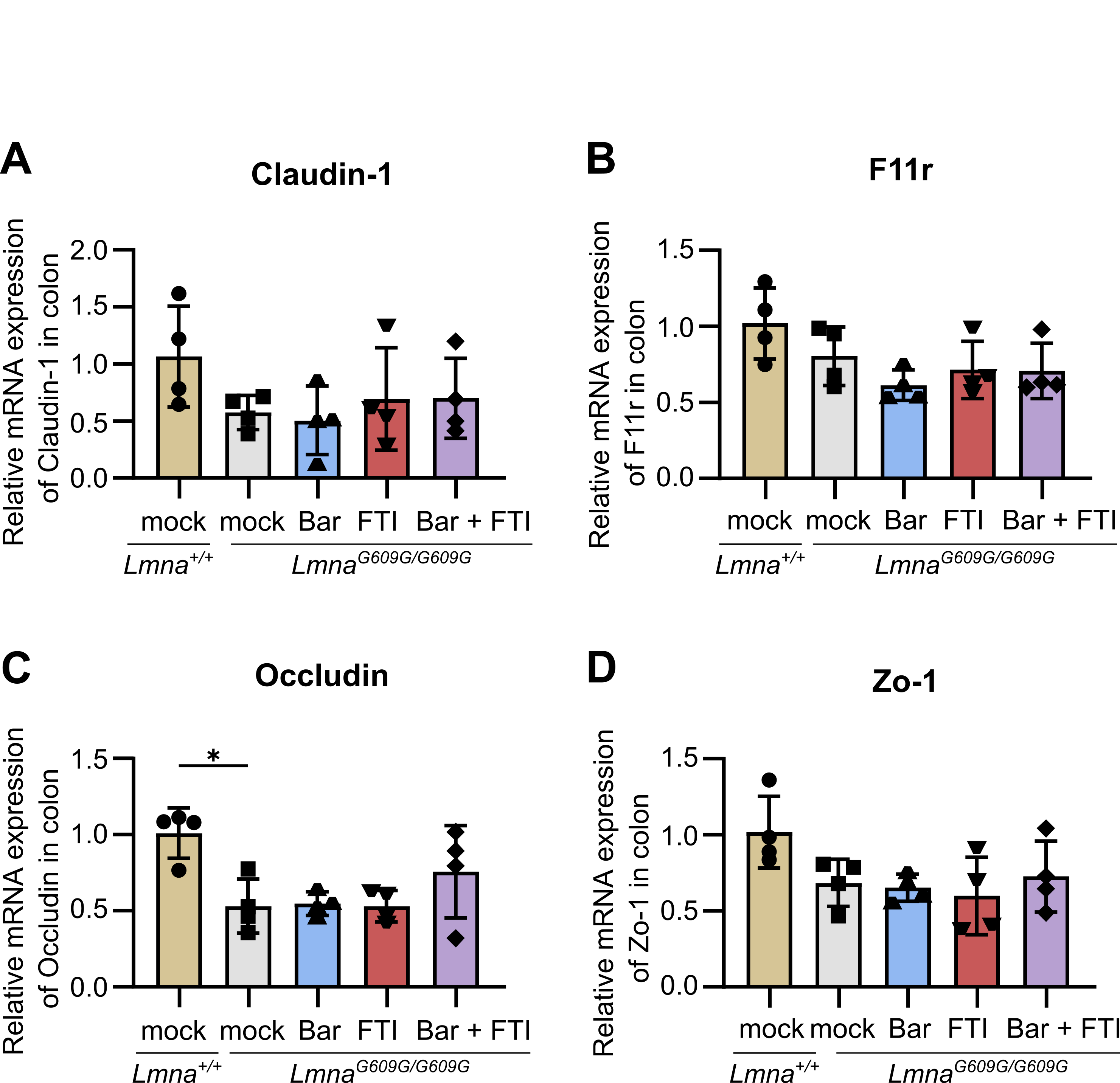


Figure S3. Analysis of epithelial barrier markers. (A-D) Analysis of mRNA levels of the tight junction markers (A) Claudin-1, (B) F11r , (C) Occludin and (D) Zo-1 in total colon extracts of *Lmna^+/+^* mock, *Lmna^G609G/G609G^* mock, Bar, FTI and Bar + FTI treated mice (n = 4). Data are expressed as the mean ± SD. *p < 0.05, calculated using ordinary one-way ANOVA followed by Tukey´s Post


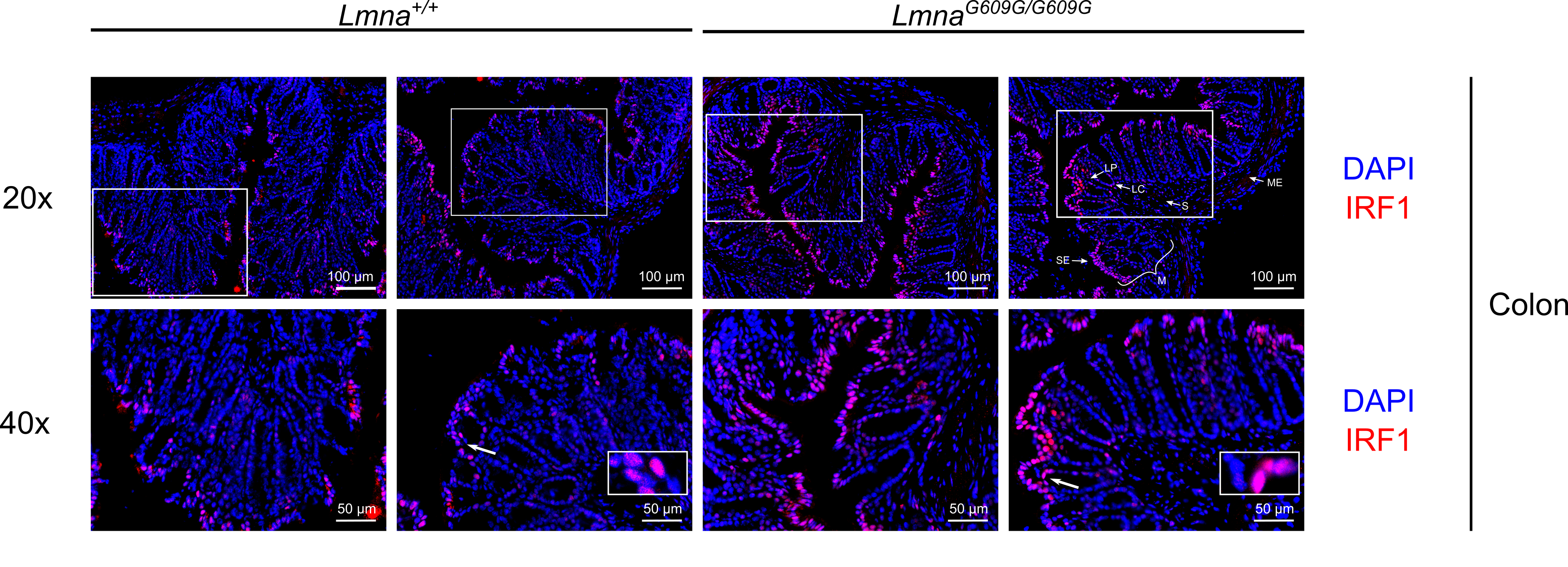
Figure S4. IRF1 expression in *Lmna^G609G/G609G^* mice. Immunofluorescence images of IRF1 (red) expression in colon tissue from *Lmna^+/+^* (left panels) and *Lmna^G609G/G609G^* mice (right panels). Nuclei were counterstained with DAPI (blue). Images were acquired at 20× magnification (scale bar, 100 µm), with boxed regions indicating the areas shown at 40× (scale bar, 50 µm). White arrows in the 40× panels indicate the areas shown in the zoom-in panels to better illustrate nuclear IRF1 staining. Abbreviations: lamina propria (LP), Lieberkühn crypt (LC), mucosa (M), muscularis externa (ME), submucosa (S), surface epithelium (SE)

**
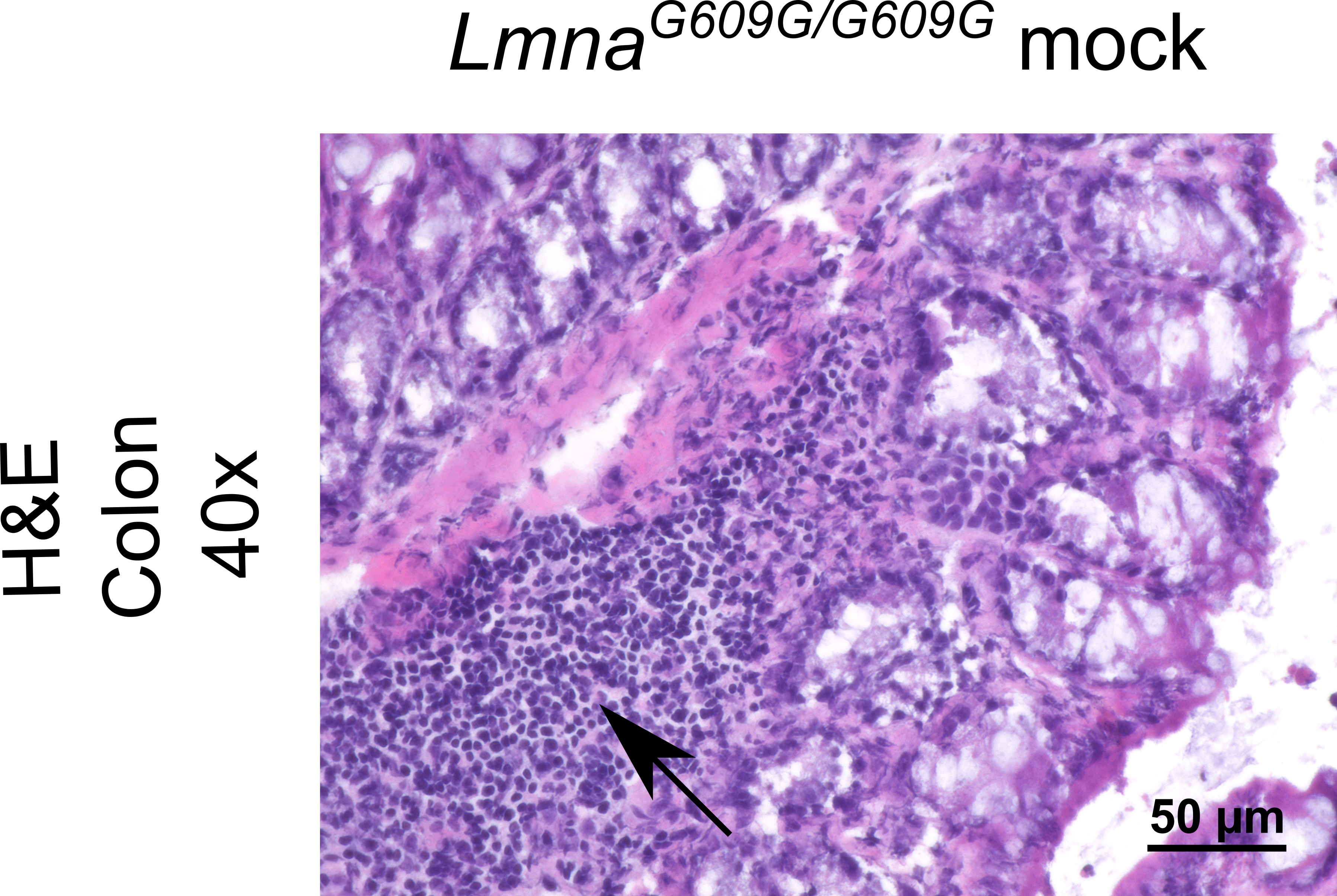
**

Figure S5. Cell infiltration in colonic tissue of *Lmna^G609G/G609G^* mock mouse. H&E stained section of colonic mucosa from a mock-treated mouse. The black arrow highlights immune cell infiltration within the mucosal layer. Scale bar: 50 µm


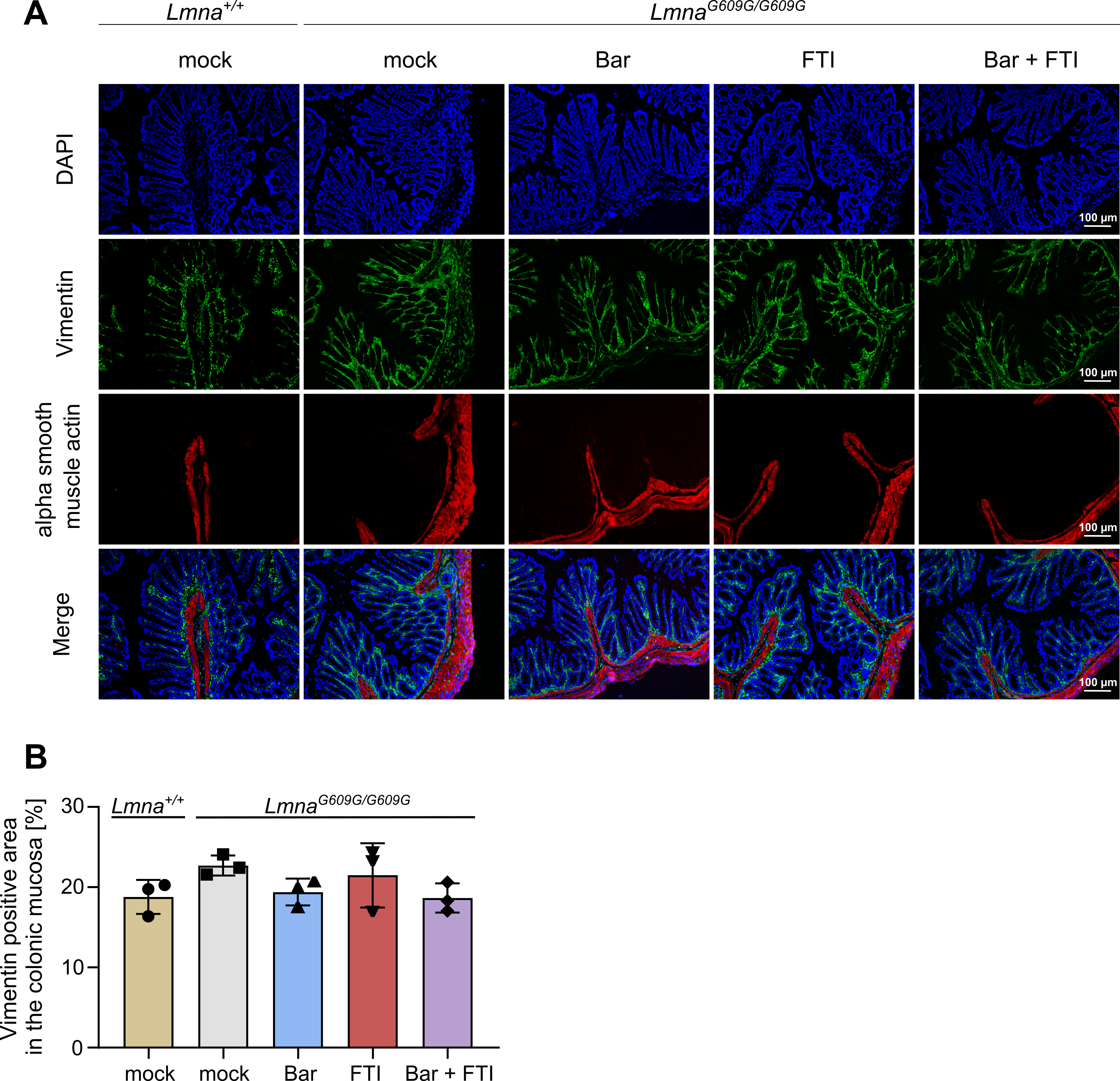


Figure S6. Vimentin expression shows an upward trend in colonic mucosa of HGPS mice. (A) Representative IF images of Vimentin (green) and alpha smooth muscle actin (red) expression in colon tissue of *Lmna^+/+^* mock, *Lmna^G609G/G609G^* mock, Bar, FTI and Bar + FTI treated mice. DAPI stain (blue) was used to counterstain nuclei (magnification 20x; scale bar 100 µm). (B) Percentage of Vimentin-stained area in the colonic mucosa based on IF analysis (n = 3). Data are expressed as the mean ± SD. Statistical analysis was calculated using ordinary one-way ANOVA


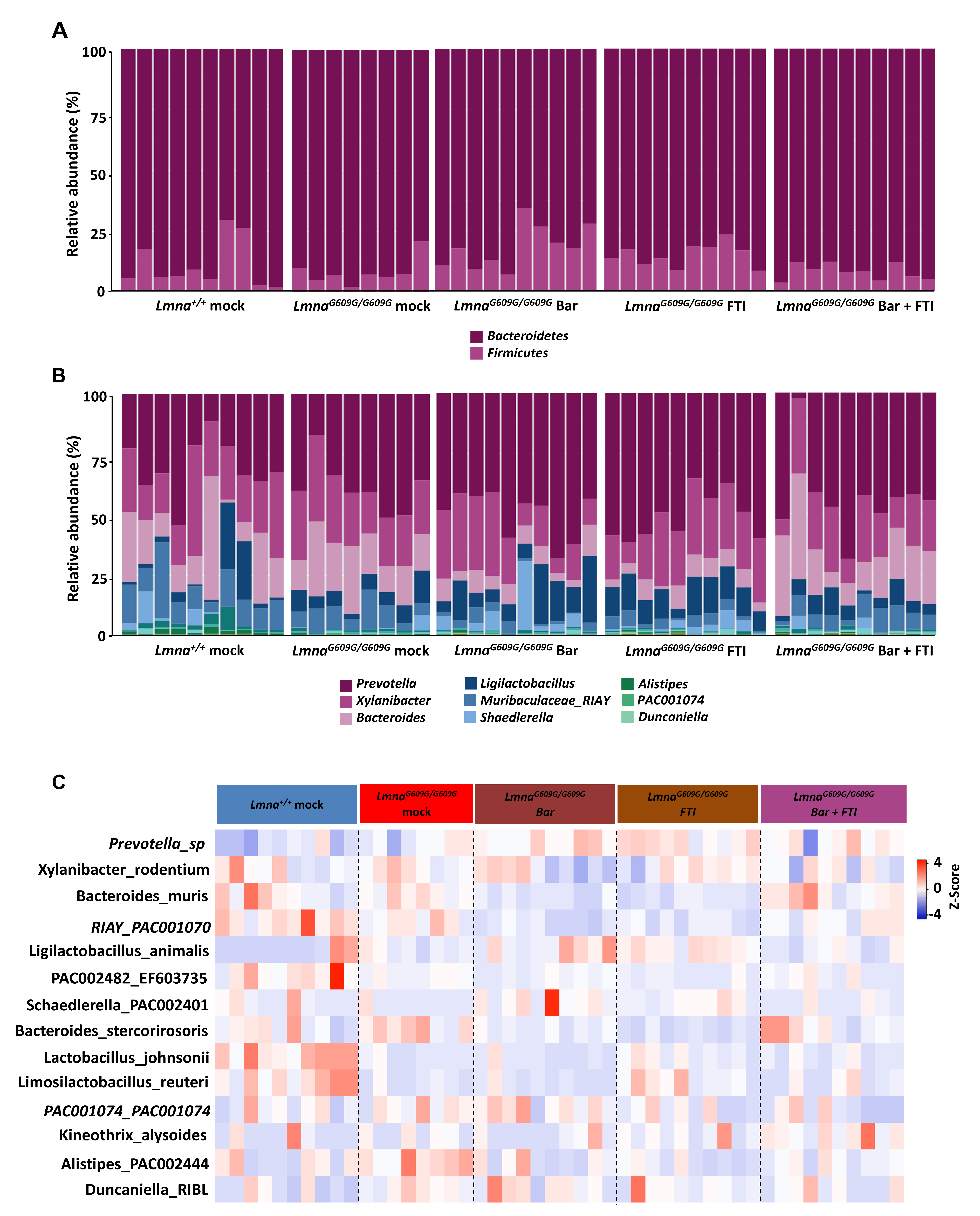
Figure S7. Relative abundances of bacteria at phylum and genera level. (A) Bar chart of taxonomy binning at phylum level of *Lmna^+/+^* mock, *Lmna^G609G/G609G^* mock, Bar, FTI and Bar + FTI treated mice. (B) Corresponding bar chart at genus level for the same groups. (C) Heat map displaying the Z-score of bacterial species across individual samples of *Lmna^+/+^* mock, *Lmna^G609G/G609G^* ^mock^ and *Lmna^G609G/G609G^* Bar, FTI and Bar + FTI treated mice. Fecal samples were collected at 90 days of age and bacterial composition was determined by summing up the relative abundances of OTUs assigned to the same phylum or genus, respectively. OTU classification was performed using the Bayesian classifier with the reference database. Sample sizes: n = 10 for *Lmna^+/+^* mock and *Lmna^G609G/G609G^* treated with Bar, FTI or Bar + FTI; n = 8 for *Lmna^G609G/G609G^* mock mice


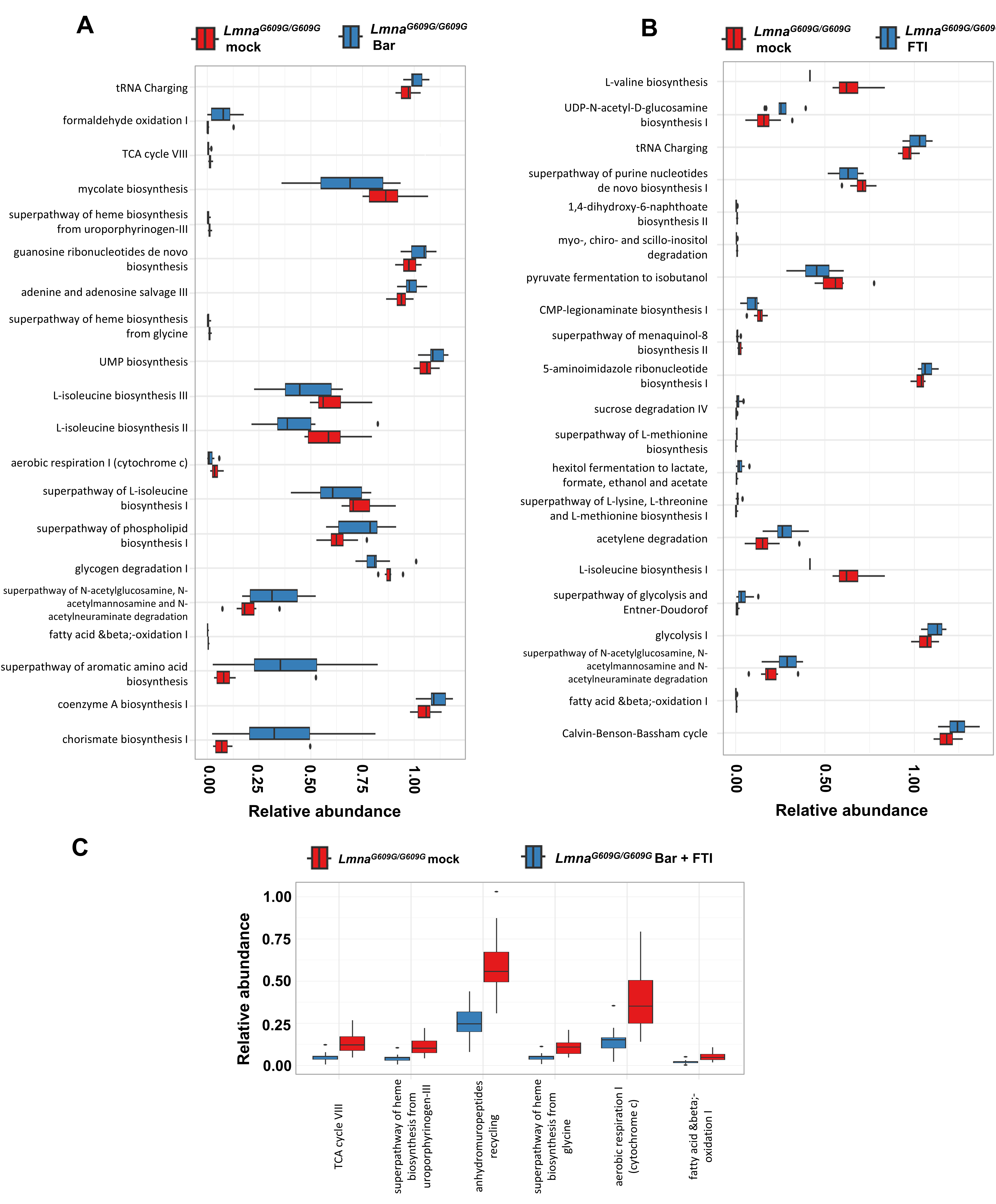
Figure S8. Predicted functional pathways regulation in gut microbiota of HGPS mice and HGPS mice treated with Bar, FTI or Bar + FTI. (A-C) PICRUSt 2 analysis tool was used to predict the gut microbiome functionality, with functional predictions mapped to the MetaCyc database. Differential abundance was assessed with DESeq2 and pathways with p < 0.05 were considered significantly different when comparing mock-treated *Lmna^G609G/G609G^* mice with (A) Bar-treated, (B) FTI-treated, or (C) Bar + FTI-treated mice. Sample sizes: n = 10 per treatment group; n = 8 mock-treated HGPS controls.


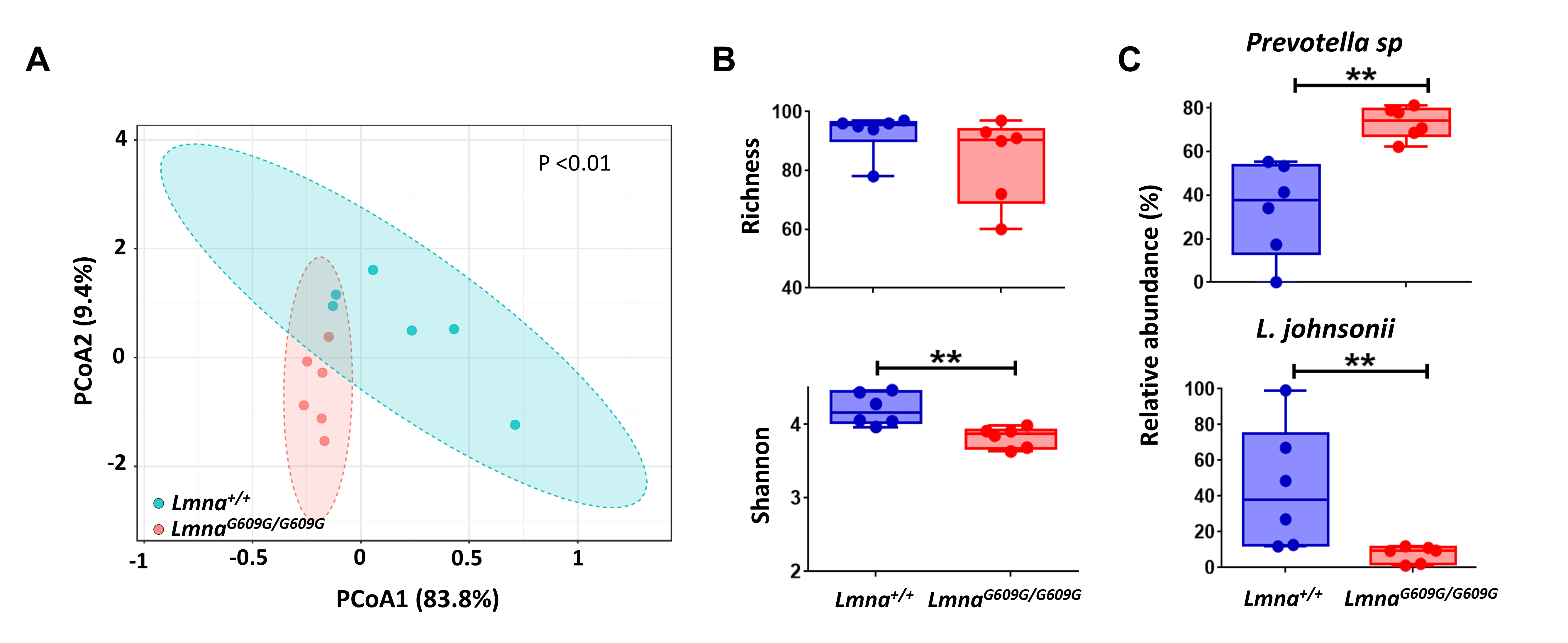


Figure S9. Gut dysbiosis in late-stage *Lmna^G609G/G609G^* mice (114 - 122 d). (A) PCoA plot of β‑diversity of gut microbiota from *Lmna^+/+^* mock and aged *Lmna^G609G/G609G^* mice. Bray-Curtis dissimilarity values were used to calculate the similarity between samples and statistical significance was tested using PERMANOVA. (B) α‑diversity measures, including species richness (number of observed OTUs) and the Shannon index, across both groups. (C) Relative abundances of dominant taxa, *Prevotella* sp. and *Lactobacillus johnsonii*, were displayed at species level in *Lmna^+/+^* mock and *Lmna^G609G/G609G^* mice. Each dot represents one mouse. Sample sizes: n = 6 per group. Fecal samples were collected on the day each *Lmna^G609G/G609G^* mouse reached its humane endpoint, as determined by a predefined health scoring system, with ages ranging from 114 to 122 d. Age-matched *Lmna^+/+^* littermates were used as controls. Statistical significance was assessed using the Wilcoxon‑Mann-Whitney tests. Asterisks indicate statistically significant differences with **p < 0.01

Supporting Information Material and Methods

### Histological Staining and Quantification

### Hematoxylin and Eosin Staining

Tissue sections were fixed in 2% paraformaldehyde (PFA) for 10 min, stained with Hematoxylin for 2 min, rinsed and treated with bluing solution for 20 sec. After two 1-min incubations in 100% ethanol, slides were briefly dipped in Eosin Y, rinsed, dehydrated through a graded ethanol series (50-100%), cleared in xylene, air-dried and mounted with synthetic resin.

### Periodic Acid-Schiff Staining

Sections were fixed in 2% PFA for 10 min, then incubated in Periodic Acid Solution (PAS) for 5 min and rinsed with highly purified water. Slides were stained with Schiff’s Reagent for 15 min, followed by a 5 min rinse under running tap water. Counterstaining was performed with Gill No. 3 Hematoxylin for 90 sec, then rinsed again. Slides were dehydrated through a graded ethanol series (50 - 100%), cleared in xylene, air-dried and mounted with synthetic resin.

### Masson´s Trichrome Staining

Tissue sections were fixed in 2% PFA for 10 min, then incubated in Bouin’s fluid overnight at room temperature. After cooling, slides were rinsed under tap water until decolorized, followed by a brief rinse in highly purified water. Sections were stained with Hematoxylin for 2 min, blued under running tap water, then incubated in Bieberich Scarlet/Acid Fuchsin for 5 min. After rinsing, slides were differentiated in 5% Phosphomolybdic/Phosphotungstic acid for 10 min, stained with Anilin Blue for 3 minutes and immersed in 0.5% acetic acid for 3 min. Slides were then dehydrated in 90% and 100% ethanol, cleared in xylene, air-dried and mounted with synthetic resin.

### β-Galactosidase Staining

Sections were fixed in 2% PFA for 10 min and washed in PBS. Slides were incubated at 37 °C for 24 h in a β-Gal staining solution containing 5 mM potassium ferricyanide, 5 mM potassium ferrocyanide, 2 mM MgCl₂, 150 mM NaCl, 0.5 mg/mL X-gal and citrate/sodium phosphate buffer. After staining, slides were washed in PBS, counterstained with nuclear fast red for 2 min, rinsed in highly purified water, air-dried and mounted with synthetic resin

### Quantification

Images were captured using the brightfield mode of the BZ-X810 microscope (Keyence) at 10x, 20x or 40x magnification. Quantification was conducted on histological images from five animals per group (n = 5) for each staining. Image analysis was conducted using ImageJ (NIH, version 1.54f). Histological parameters from H&E-stained sections were assessed by taking 50 independent measurements per mouse across multiple regions of the distal colon to ensure representative assessment. Measurements included muscularis mucosa thickness and crypt depth (measured from the base of the crypt of Lieberkühn to the luminal surface). All measurements were assessed in µm, using the calibrated scale function in ImageJ. Goblet cells were visualized using PAS staining. For quantification, the number of goblet cells was manually counted in 50 crypts of Lieberkühn per mouse. Results are reported as the average number of goblet cells per crypt. For Masson’s Trichrome staining, ten images per mouse were acquired at 40x magnification. The exposure time was fixed at 1/60 sec to ensure consistency across all samples. To isolate collagen-specific signals, images were processed using the “Colour Deconvolution2” plugin with the Masson Trichrome vector set, which separates blue-stained components from red-stained components. Thresholding was applied to both channels (blue: 0-190; red: 0-80. To reduce false-positive collagen detection caused by hematoxylin-stained nuclei appearing in both channels, the red signal was subtracted from the blue using the “Image Calculator” function. The resulting image was converted into a binary mask. Collagen content was quantified by measuring the collagen-positive area within manually defined ROIs corresponding to the mucosal layer. The percentage of collagen-positive area was calculated relative to the total mucosal area for each image. To quantify β-Gal–positive cells, ten regions of interest were selected from representative images of the distal colon per mouse, acquired at 20x magnification with a fixed exposure time of 1/250 sec to ensure consistency across samples. The number of blue-stained β-Gal-positive cells was quantified relative to the total number of nuclei per image. The “H PAS” vector in the “Colour Deconvolution2” plugin was used, as it effectively separated the deep blue precipitate from X-gal staining (channel 1) from the pink nuclear counterstain (channel 2). After deconvolution, both channels were thresholded (channel 1: 0-100; channel 2: 0-215) and converted to binary masks. A watershed function was applied to improve cell separation, followed by particle analysis to count individual stained nuclei. The total number of blue-stained cells was then divided by the total number of nuclei to calculate the percentage of β-Gal-positive cells.

### Immunofluorescence

### Quantification

Images were captured on a BZ-X810 fluorescence microscope (Keyence) using 20x or 40x objectives. For each marker, the exposure time was fixed across all samples to ensure consistency. Quantification used images from three animals per group (n = 3) for each staining. Image analysis was performed in ImageJ/Fiji (NIH, v1.54f). For PAI-1 quantification, five non-overlapping mucosal regions were selected per mice. DAPI-positive nuclei were counted in the DAPI channel alone by applying a fixed threshold (25-255), converting to a binary mask, separating touching nuclei with the watershed function and using the “Analyze Particles” function. A cell was considered PAI-1-positive if a distinct red signal was observed co-localizing with a DAPI-stained nucleus in the merged image. For Ki67 and Lgr5 quantification, eight ROIs per sample were analyzed. Ki67-positive nuclei were quantified using the same automated particle analysis method as for DAPI, while Lgr5-positive cells were counted manually in merged images using the same visual criteria as for PAI-1. For vimentin quantification, five non-overlapping ROIs per sample were analyzed after converting images to 8-bit, applying a fixed threshold (10-255), converting to a mask and measuring the positive area within each ROI. For all cell-based markers (PAI-1, Ki67, Lgr5), the number of positive cells in each ROI was normalized to the total number of DAPI-positive nuclei to yield the percentage of positive cells per ROI. For vimentin, the positive staining area within each ROI was measured and expressed as a percentage of the total ROI area.
